# Loss-of-function mutations in *ATP6AP1* and *ATP6AP2* in granular cell tumors

**DOI:** 10.1038/s41467-018-05886-y

**Published:** 2018-08-30

**Authors:** Fresia Pareja, Alissa H. Brandes, Thais Basili, Pier Selenica, Felipe C. Geyer, Dan Fan, Arnaud Da Cruz Paula, Rahul Kumar, David N. Brown, Rodrigo Gularte-Mérida, Barbara Alemar, Rui Bi, Raymond S. Lim, Ino de Bruijn, Sho Fujisawa, Rui Gardner, Elvin Feng, Anqi Li, Edaise M. da Silva, John R. Lozada, Pedro Blecua, Leona Cohen-Gould, Achim A. Jungbluth, Emad A. Rakha, Ian O. Ellis, Maria I. A. Edelweiss, Juan Palazzo, Larry Norton, Travis Hollmann, Marcia Edelweiss, Brian P. Rubin, Britta Weigelt, Jorge S. Reis-Filho

**Affiliations:** 1 Department of Pathology, Memorial Sloan Kettering Cancer Center, New York, 10065 NY USA; 2 Genetics and Molecular Biology, Federal University of Rio Grande do Sul, Porto Alegre, 91501-970 Brazil; 3 Department of Pathology, Fudan University Shanghai Cancer Center, Shanghai, 200032 China; 4 Molecular Cytology Core Facility, Memorial Sloan Kettering Cancer Center, New York, 10065 NY USA; 5 Flow Cytometry Core Facility, Memorial Sloan Kettering Cancer Center, New York, 10065 NY USA; 6 Department of Radiation Oncology, Memorial Sloan Kettering Cancer Center, New York, 10065 NY USA; 7 Department of Biochemistry, Weill Cornell Medical College, New York, 10065 NY USA; 8 Department of Pathology, University of Nottingham, Nottingham, NG7 2RD UK; 9 Hospital de Clínicas, Federal University of Rio Grande do Sul, Porto Alegre, 90035-903 Brazil; 10 Department of Pathology, Jefferson Medical College, Philadelphia, 19107 PA USA; 11 Department of Medicine, Memorial Sloan Kettering Cancer Center, New York, 10065 NY USA; 12 Departments of Pathology and Cancer Biology, Robert J. Tomsich Pathology and Laboratory Medicine Institute and The Lerner Research Institute, Cleveland Clinic, Cleveland, 44195 OH USA

## Abstract

Granular cell tumors (GCTs) are rare tumors that can arise in multiple anatomical locations, and are characterized by abundant intracytoplasmic granules. The genetic drivers of GCTs are currently unknown. Here, we apply whole-exome sequencing and targeted sequencing analysis to reveal mutually exclusive, clonal, inactivating somatic mutations in the endosomal pH regulators *ATP6AP1* or *ATP6AP2* in 72% of GCTs. Silencing of these genes in vitro results in impaired vesicle acidification, redistribution of endosomal compartments, and accumulation of intracytoplasmic granules, recapitulating the cardinal phenotypic characteristics of GCTs and providing a novel genotypic–phenotypic correlation. In addition, depletion of ATP6AP1 or ATP6AP2 results in the acquisition of oncogenic properties. Our results demonstrate that inactivating mutations of *ATP6AP1* and *ATP6AP2* are likely oncogenic drivers of GCTs and underpin the genesis of the intracytoplasmic granules that characterize them, providing a genetic link between endosomal pH regulation and tumorigenesis.

## Abstract (web summary)

Granular cell tumors (GCTs) are rare tumors that arise in multiple anatomical locations. Here, the authors investigate the genomics of GCTs, finding inactivating somatic mutations in *ATP6AP1* or *ATP6AP2* in 72% of the 82 GCTs analyzed. In vitro manipulation of these genes recapitulated GCT phenotypes in cellular models.

## Introduction

Granular cell tumors (GCTs) are uncommon neoplasms which can arise in multiple anatomical sites. These tumors usually follow a benign course^1^, but may occasionally exhibit an aggressive behavior with local and distant recurrences^1–3^. GCTs are characterized by abundant intracytoplasmic granules, whose nature and function remain unclear^1^. The genetic landscape of GCTs and the mechanisms underpinning the presence of their characteristic intracytoplasmic granules are currently unknown^1^.

There is a burgeoning body of evidence indicating that genetic analysis of rare cancer types may provide unique opportunities for the identification of novel cancer drivers^4^. A subset of rare tumors not uncommonly have simple genomes, with a paucity of copy number alterations (CNAs) and somatic mutations, and are characterized by highly recurrent, specific, or even pathognomonic, somatic mutations, or fusion genes^4^. These tumors have distinctive phenotypes and often arise in diverse anatomic locations. Akin to these tumors, GCTs are rare, arise in different anatomic locations, and display peculiar morphologic features; hence, we posited that they could also be underpinned by a highly recurrent genetic alteration.

Here, through a whole-exome sequencing (WES) and targeted sequencing analysis of GCTs, we uncovered highly recurrent and mutually exclusive inactivating mutations targeting the endosomal pH regulators *ATP6AP1* and *ATP6AP2* in GCTs. In vitro silencing of ATP6AP1 and ATP6AP2 in human Schwann cells and epithelial cells resulted in the accumulation of intracytoplasmic granules that are ultra-structurally and phenotypically similar to those of human GCTs, altered endosomal acidification and oncogenic properties, thereby establishing a novel genotypic–phenotypic correlation.

## Results

### Recurrent *ATP6AP1* and *ATP6AP2* somatic mutations in GCTs

GCTs were retrieved from the authors’ institutions, following the approval of this study by the local research ethics committees or institutional review boards (IRBs) of the contributing authors’ institutions. Patient consent was obtained where appropriate, according to the protocols approved. Upon central pathology review, 82 cases were classified as GCTs, which originated in different anatomic locations, including skin (*n* = 40), soft tissue (*n* = 15), gastrointestinal tract (*n* = 13), breast (*n* = 6), tongue (*n* = 5), and other locations (*n* = 3; Supplementary Table 1).

**Fig. 1:**
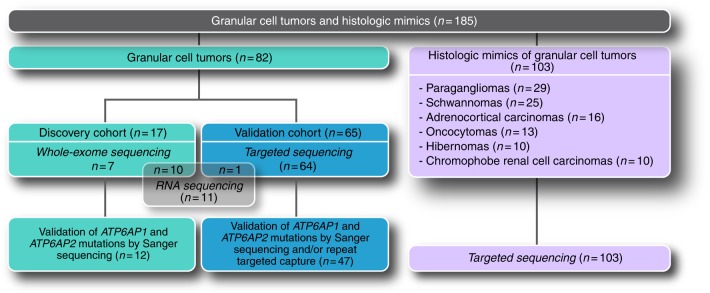
Schematic representation of the tissue samples and sequencing methods employed in this study. Depiction of the discovery and validation cohorts of granular cell tumors, and the series of histologic mimics of these tumors included in this study, and of the sequencing analysis methods utilized

**Fig. 2:**
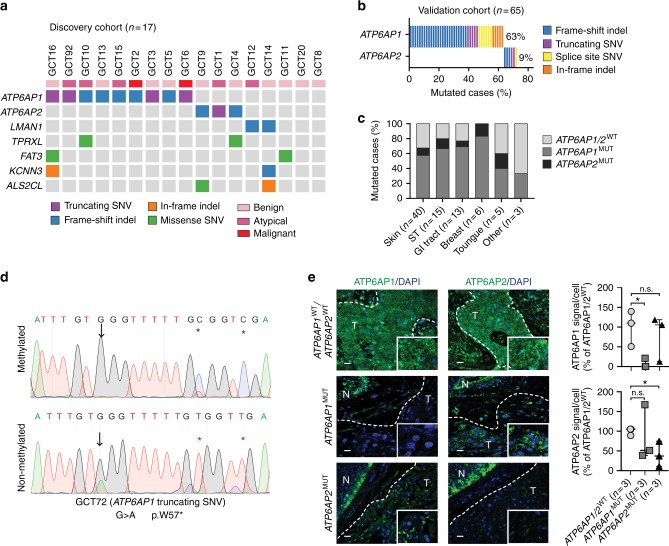
Inactivating *ATP6AP1* and *ATP6AP2* somatic mutations are highly prevalent in granular cell tumors. **a** Recurrent non-synonymous somatic mutations identified in granular cell tumors (GCTs) by whole-exome sequencing (*n* = 17). Cases are shown in columns and genes in rows. The histologic classification following the Fanburg-Smith criteria is shown in the phenotype bar (top). Mutation types are color-coded according to the legend. SNV, single-nucleotide variation. **b** Mutation frequencies of *ATP6AP1* and *ATP6AP2* identified by targeted capture sequencing of additional GCTs of the validation cohort (*n* = 65). Mutation types are color-coded according to the legend. **c** Frequency of *ATP6AP1* and *ATP6AP2* mutations according to anatomical location. The *ATP6AP1* and *ATP6AP2* mutational status is color-coded according to the legend. GI, gastrointestinal; ST, soft tissue. **d** Representative Sanger sequencing electropherograms of bisulfite analysis of an *ATP6AP1*-mutated GCT in a female patient. Arrows highlight the altered nucleotide. *Cytosine converted to thymidine upon bisulfite treatment. **e** ATP6AP1 and ATP6AP2 protein expression in *ATP6AP1*-mutated, *ATP6AP2*-mutated, and *ATP6AP1*- and *ATP6AP2*-wild-type GCTs assessed by immunofluorescence (*n* = 3). Tumor borders are depicted by a dashed line; T, tumor; N, normal stromal/epithelial cells as internal controls. Scale bars, 20 μm. Quantification of ATP6AP1 and ATP6AP2 fluorescent signal/ cell (*n* = 3; mean ± S.D.); n.s. = non significant, *P < 0.05; two-tailed unpaired *t*-tests. MUT, mutated; WT, wild type. Experiments in (**d**, **e**) were independently performed at least three times

Our study comprised a discovery cohort (*n* = 17) and a validation cohort (*n* = 65; Fig. 1). To determine whether GCTs would be underpinned by a pathognomonic genetic alteration, we subjected the 17 GCTs of the discovery cohort to WES and 11 GCTs to RNA sequencing, with 10 cases being subjected to both (Fig. 1, Supplementary Table 1). These analyses revealed a paucity of CNAs (Supplementary Fig. 1a), no recurrently mutated known cancer genes, and no recurrent likely pathogenic fusion genes (Fig. 2a, Supplementary Table 2). Rather, WES analysis revealed recurrent, mutually exclusive, and clonal loss-of-function (i.e., nonsense or frameshift) somatic mutations affecting *ATP6AP1* or *ATP6AP2* in 12/17 of the GCTs analyzed (*p* = 0.041, CoMEt; Fig. 2a, Supplementary Fig. 1b, Supplementary Tables 3–4). All *ATP6AP1* and *ATP6AP2* somatic mutations identified by WES were validated by Sanger sequencing (Supplementary Fig. 1c). To validate our findings, we subjected 65 additional GCTs from the validation cohort to targeted massively parallel sequencing, which revealed mutually exclusive loss-of-function mutations (i.e., nonsense, frameshift, or splice-site) affecting *ATP6AP1* and *ATP6AP2* in 36/65 and 6/65 cases, respectively (*p* = 0.0028,CoMEt; Figs. 1 and 2b, Supplementary Table 1). In addition, 5/65 GCTs harbored *ATP6AP1* in-frame indels affecting evolutionarily conserved residues (Fig. 2b, Supplementary Fig. 1d). All *ATP6AP1* and *ATP6AP2* mutations identified by targeted sequencing (*n* = 47) were validated by Sanger sequencing and/or repeat targeted capture sequencing analysis (Fig. 1a and Supplementary Table 1). In total, 72% (59/82) of the GCTs analyzed here harbored likely inactivating somatic mutations in *ATP6AP1* or *ATP6AP2*. Of note, no differences in histologic features (Supplementary Table 5) or anatomical site (Fig. 2c) of GCTs according to the *ATP6AP1*/*ATP6AP2* mutational status were observed.

### *ATP6AP1* and *ATP6AP2* inactivating mutations are expressed

*ATP6AP1* and *ATP6AP2* map to Xq28 and Xp11.4, respectively. Therefore, a single inactivating mutation in either gene targeting the X chromosome in males or the active/non-methylated X chromosome in females would be sufficient to cause its complete loss of function^5^. To determine whether *ATP6AP1* and *ATP6AP2* loss-of-function mutations affect the active/non-methylated X chromosome in females, we conducted bisulfite sequencing of GCTs harboring *ATP6AP1* mutations in the vicinity of CpG islands. No GCTs included in this study harbored *ATP6AP2* mutations adjacent to CpG islands. Bisulfite sequencing revealed that the *ATP6AP1* mutations tested were present in non-methylated DNA, indicating that these mutations affected the active/non-methylated X chromosome of GCTs in females (Fig. 2d and Supplementary Fig. 2a). To validate these findings using an orthogonal approach, we performed a modified human androgen receptor (HUMARA) assay following DNA restriction digestion with the methylation-sensitive restriction enzyme *Hha*I, which only cleaves non-methylated DNA. These assays showed that mock-digested DNA displayed *ATP6AP1* mutations, whereas DNA following treatment with *Hha*I was wild-type (Supplementary Fig. 2b). These findings indicate that *ATP6AP1* mutations affect the active X chromosome in females.

Next, we sought to determine whether loss-of-function mutations affecting *ATP6AP1* and *ATP6AP2* result in their decreased expression. Messenger RNA (mRNA) expression of *ATP6AP1-* and *ATP6AP2*-mutant forms was detected in all *ATP6AP1-* and *ATP6AP2-*mutant GCTs analyzed by RNA sequencing and/or complementary DNA (cDNA) sequencing, respectively (Supplementary Figs. 2c, d). We then aimed to determine whether *ATP6AP1* and *ATP6AP2* loss-of-function mutations result in reduced protein expression. As expected, immunofluorescence and western blot analyses revealed lower ATP6AP1 and ATP6AP2 protein levels in GCTs harboring *ATP6AP1* or *ATP6AP2* mutations, respectively, than in GCTs wild-type for these genes and in normal tissues (Fig. 2e and Supplementary Figs. 3a, b). Taken together, these data demonstrate that inactivating somatic mutations affecting *ATP6AP1* and *ATP6AP2* are expressed and result in a significant reduction of their respective protein levels.

### *ATP6AP1/ATP6AP2* mutations are likely pathognomonic for GCTs

**Fig. 3:**
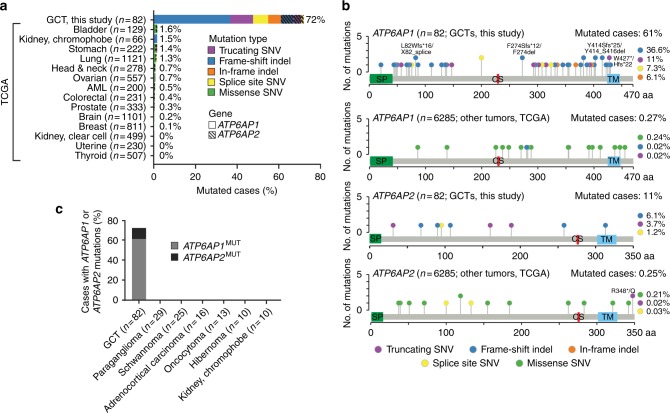
Mutations affecting *ATP6AP1* and *ATP6AP2* are likely pathognomonic for granular cell tumors. **a** Frequency and type of *ATP6AP1* and *ATP6AP2* mutations in 82 granular cell tumors (GCTs) from this study and in 6285 non-hypermutated cancers from The Cancer Genome Atlas (TCGA) including samples from 16 common cancer types. AML, acute myeloid leukemia; SNV, single-nucleotide variant. **b** Schematic representation of ATP6AP1 and ATP6AP2 protein domains depicting the 59 mutations affecting *ATP6AP1* and *ATP6AP2* in 82 GCTs from this study and 33 mutations affecting these genes in 6285 non-hypermutated common cancers from TCGA. Mutations are shown on the *x*-axis, and the frequency of a particular mutation is represented by the height of each 'lollipop' (*y*-axis). Mutation types are color-coded according to the legend. **c** *ATP6AP1* and *ATP6AP2* mutational frequency in 82 GCTs and 103 histologic mimics sequenced in this study. Indel, small insertion and deletion; MUT, mutant; SNV, single-nucleotide variant; WT, wild-type

To determine whether *ATP6AP1* and *ATP6AP2* loss-of-function mutations are pathognomonic for GCTs, we investigated their presence in 6285 non-hypermutated cancers across 14 common cancer types from The Cancer Genome Atlas (TCGA) studies retrieved from the cBioPortal^6^. In contrast to the high frequency of likely inactivating *ATP6AP1* (61%) and *ATP6AP2* (11%) somatic mutations in the GCTs analyzed in this study, mutations affecting these genes were found in only 0.27% and 0.25% of common cancers, respectively (Fig. 3a, b). Moreover, at variance with GCTs, where *ATP6AP1* and *ATP6AP2* mutations were predominantly frameshift or nonsense, consistent with the mutational spectra of potential tumor suppressor genes, inactivating mutations targeting *ATP6AP1* and *ATP6AP2* were found to be vanishingly rare in common cancers from TCGA, with a prevalence of 0.03% and 0.05% of cases, respectively (Fig. 3b).

We next sequenced the entire coding region of *ATP6AP1* and *ATP6AP2* in 103 histologic mimics of GCTs, including paragangliomas (*n* = 29), schwannomas (*n* = 25), adrenocortical carcinomas (*n* = 16), oncocytomas (*n* = 13), hibernomas (*n* = 10), and chromophobe renal carcinomas (*n* = 10). These are tumors that may occasionally display intracytoplasmic granules and may be considered in the differential diagnosis of GCTs (Fig. 1). This analysis did not reveal any inactivating somatic mutations affecting either of these genes in the 103 tumors analyzed (Fig. 3c).

Taken together, these findings provide strong circumstantial evidence to demonstrate that *ATP6AP1* and *ATP6AP2* loss-of-function mutations are pathognomonic for GCTs.

### *ATP6AP1* and *ATP6AP2* inactivation drives the GCT phenotype

**Fig. 4:**
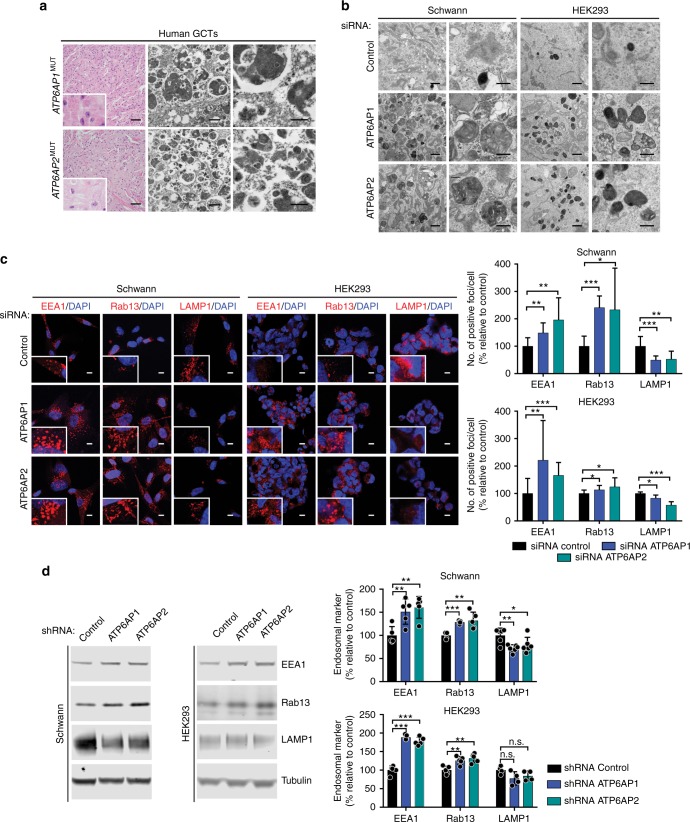
ATP6AP1 and ATP6AP2 loss of function results in a granular phenotype and redistribution of endosomal compartments. **a** Representative hematoxylin-and-eosin micrographs and transmission electron micrographs of human granular cell tumors (GCTs) harboring *ATP6AP1* or *ATP6AP2* loss-of-function mutations. Scale bars, 50 μm (left), 1 μm (center), and 0.5 μm (right). **b** Representative transmission electron micrographs of primary Schwann cells and HEK293 cells transfected with validated short-interfering RNAs (siRNAs) targeting ATP6AP1, ATP6AP2, or non-targeting control. Scale bars, 1 μm (left) and 0.5 μm (right). **c** Representative confocal micrographs of immunofluorescence analysis of EEA1, Rab13, and LAMP1 (red) and 4-6-diamidino-2-phenylindole (DAPI, blue) expression in primary Schwann cells and HEK293 cells transfected with siRNAs against ATP6AP1, ATP6AP2, or control. Scale bars, 10 μm. Quantification (right) of EEA1-, Rab13-, and LAMP1-positive foci/cell relative to control. Error bars, mean ± S.D. (*n* = 10). *P < 0.05, **P < 0.01, ***P < 0.001; two-tailed unpaired *t*-test. **d** Representative western blot analysis of EEA1, Rab13, and LAMP1 protein levels in immortalized Schwann cells and HEK293 cells with stable knockdown of ATP6AP1, ATP6AP2, or control. Tubulin was used as protein loading control. Quantification (right) of EEA1, Rab13, and LAMP1 protein levels relative to control. Experiments were performed in replicates (Schwann cells, *n* ≥ 5; HEK293 cells, *n* ≥ 4). Error bars, mean ± S.D.; n.s., non significant; *P < 0.05, **P < 0.01, ***P < 0.001; two-tailed unpaired *t*-test

*ATP6AP1* and *ATP6AP2* encode for regulatory subunits of the vacuolar H^+^-ATPase (V-ATPase) complex, a key regulator of intracellular organelle pH^7^, and play a role in the control of endosomal acidification and vesicle trafficking^8–10^. We posited that loss of function of ATP6AP1 and ATP6AP2 would lead to altered endocytosis resulting in the presence of the intracytoplasmic granules characteristic of GCTs. Depletion of ATP6AP1 or ATP6AP2 in primary Schwann cells, a putative cell of origin of GCTs^1^, resulted in the accumulation of numerous intracytoplasmic structures of heterogeneous morphology with electron-dense and -clear contents, remarkably similar to those observed in the human GCTs we analyzed (Fig. 4a, b). A similar but less overt phenotype was observed upon ATP6AP1 or ATP6AP2 silencing in HEK293 cells and MCF-10A cells (Fig. 4b, Supplementary Fig. 4a, b).

ATP6AP1 and ATP6AP2 are required for endosomal acidification^8,9^, which is essential for proper endocytic flux^7^. Immunofluorescence analysis following silencing of either gene demonstrated an altered distribution of endosomal compartments, due to accumulation of early endosomes (EEA1-positive) and recycling endosomes (Rab13-positive), which are endosomal compartments with a higher pH^11^. In addition, silencing of ATP6AP1 or ATP6AP2 led to a decreased number of lysosomes (LAMP1-positive; Fig. 4c). Consistent with these findings, we observed increased EEA1 and Rab13 protein levels, coupled with decreased LAMP1 expression upon stable silencing of ATP6AP1 or ATP6AP2 in immortalized Schwann cells and HEK293 cells by western blot analysis (Fig. 4d and Supplementary Fig. 4c). Overall, these data suggest that the characteristic intracytoplasmic granules of GCTs might be due to an abnormal distribution of endosomal compartments resulting in accumulation of those with a higher pH.

### Depletion of ATP6AP1 or ATP6AP2 and endosomal acidification

**Fig. 5:**
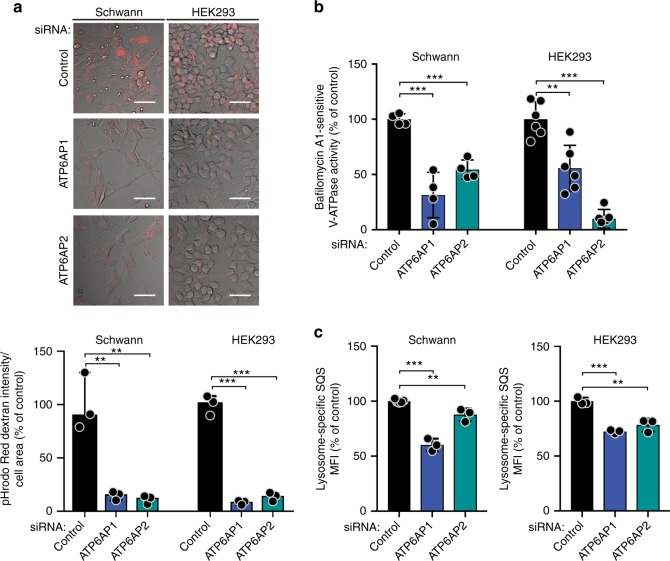
ATP6AP1 and ATP6AP2 loss of function leads to decreased acidification of endosomal compartments and V-ATPase activity. **a** Representative confocal pHrodo Red dextran fluorescence micrographs of primary Schwann cells and HEK293 cells transfected with siRNAs against ATP6AP1, ATP6AP2, or control. Scale bars, 50 μm. Quantification (bottom) of pHrodo Red dextran fluorescence/cell area relative to control. Error bars, mean ± S.D. (*n* = 3). **P < 0.01, ***P < 0.001; two-tailed unpaired *t*-test. **b** Enzymatic V-ATPase activity assay in immortalized Schwann cells and HEK293 cells with stable silencing of ATP6AP1 or ATP6AP2 and control cells, in the presence of Bafilomycin-A1. V-ATPase activity is depicted compared to control. Error bars, mean ± S.D. Schwann cells, *n* = 4; HEK293 cells, *n* = 6. **P < 0.01, ***P < 0.001; two-tailed unpaired *t*-test. **c** Lysosomal activity assay of primary Schwann cells and HEK293 cells transfected with siRNAs against ATP6AP1, ATP6AP2, or control. Median fluorescence intensity (MFI) of the lysosome-specific self-quenched substrate (SQS) fluorescent signal as determined by flow cytometry, compared to control. Error bars, mean ± S.D. Schwann cells, *n* ≥ 3; HEK293 cells, *n* = 3. **P < 0.01, ***P < 0.001; two-tailed unpaired *t*-test

Regulation of endosomal pH is key for the adequate functioning of endocytosis^12^. Given that ATP6AP1 and ATP6AP2 are integral components of the V-ATPase complex^7^, we posited that their loss of function would result in suboptimal acidification of endosomal compartments, coupled with an altered endocytic flux. We sought to determine the effect of ATP6AP1 and ATP6AP2 depletion on the acidification of endocytic organelles. Live-cell microscopy experiments using the pH-sensitive pHrodo Red dextran, which displays increased fluorescence with decreasing pH, revealed that transient or stable silencing of ATP6AP1 or ATP6AP2 in Schwann cells and HEK293 cells resulted in decreased pHrodo Red dextran fluorescence, indicative of reduced acidification of endosomal compartments (Fig. 5a and Supplementary Fig. 4d). Although the effect on endosomal acidification upon simultaneous transient silencing of ATP6AP1 and ATP6AP2 was more pronounced than following silencing of either gene separately (Supplementary Fig. 4e), the extent of redistribution of endosomal compartments upon single and double silencing of these genes was comparable (Supplementary Fig. 4f).

We posited that the decreased endosomal acidification observed following ATP6AP1 or ATP6AP2 silencing would be due to abnormal functioning of the V-ATPase. Hence, we performed a V-ATPase activity assay using the membrane fraction of immortalized Schwann cells and HEK293 cells, where ATP6AP1 or ATP6AP2 had been stably silenced, and control cells, in the presence and absence of Bafilomycin-A1 (a selective inhibitor of the V-ATPase). These experiments revealed that silencing of ATP6AP1 or ATP6AP2 led to decreased V-ATPase activity compared to control (Fig. 5b). To corroborate these findings, we evaluated the impact of loss of function of these genes on lysosomal activity, as lysosomes are acidic endosomal compartments. Upon transient silencing of ATP6AP1 or ATP6AP2 in Schwann cells and HEK293 cells, we observed decreased lysosomal activity, detected as reduced fluorescence of the lysosome-specific self-quenched substrate, which acts as endocytic cargo generating fluorescent signal upon lysosomal degradation. These findings provide further evidence that ATP6AP1 and ATP6AP2 loss of function results in abnormal functioning of the V-ATPase, with a subsequent decrease in lysosomes and lysosomal activity (Fig. 5c and Supplementary Fig. 4g).

We next investigated whether loss of function of ATP6AP1 or ATP6AP2 would result in a general alteration of endocytic flux, through the evaluation of the trafficking of transferrin, which undergoes receptor-mediated endocytosis. We observed that transient and stable silencing of ATP6AP1 or ATP6AP2 led to decreased delivery of pHrodo Red transferrin to acidic endosomal compartments, suggesting that depletion of either gene results in a block of endocytic flux (Supplementary Figs. 4h, i).

The activity of the V-ATPase is regulated by several factors, such as the assembly of its cytoplasmic V1 domain, which hydrolyzes adenosine triphosphate, and its membrane-bound V0 domain, which translocates protons across endosomal membranes^7^. We sought to determine whether loss of function of ATP6AP1 or ATP6AP2 would result in decreased V-ATPase activity by affecting the assembly of the V1 and V0 domains. Hence, we evaluated the protein levels of ATP6V1A and ATP6V0D1, subunits of the V-ATPase V1 and V0 domains, respectively, in the membrane and cytoplasmic fractions of immortalized Schwann cells and HEK293 cells where ATP6AP1 or ATP6AP2 had been stably silenced, and in control cells. Given that the V1 domain is cytoplasmic, membranous ATP6V1A could be regarded as a surrogate marker for the relative abundance of assembled V-ATPase. Silencing of ATP6AP1 or ATP6AP2 resulted in decreased membrane/cytosolic ATP6V1A (Supplementary Fig. 5a), indicating that loss of function of ATP6AP1 or ATP6AP2 is associated with a defective assembly of the V0 and V1 domains of the V-ATPase. Interestingly, we also observed that silencing of either gene caused reduced total ATP6V0D1 levels, suggesting that loss of function of ATP6AP1 or ATP6AP2 leads to a decreased biogenesis or stability of the V-ATPase V0 domain (Supplementary Fig. 5a), providing a putative mechanism for the observed decrease in lysosomal acidification upon depletion of either gene. Consistent with these findings, we observed a mild increase in the levels of LC3B-II upon ATP6AP1 or ATP6AP2 silencing in immortalized Schwann cells and HEK293 cells at baseline conditions compared to control (Supplementary Fig. 5b), indicating that loss of function of either gene results in a modest but significant impairment of the autophagic flux.

Taken together, our findings provide a likely mechanistic basis for the characteristic intracytoplasmic granules of GCTs, given that ATP6AP1 or ATP6AP2 silencing impairs the assembly of the V-ATPase and vesicular acidification, resulting in abnormal endocytic flux, which might be accountable for the accumulation of endosomal vesicles with a higher pH.

### Oncogenic properties of *ATP6AP1* or *ATP6AP2* inactivation

In light of the high frequency of *ATP6AP1* and *ATP6AP2* inactivating mutations in GCTs, which suggests a putative tumor suppressor role for these genes, we hypothesized that their loss of function would result in the acquisition of oncogenic properties in vitro in GCT-relevant cell models. While stable silencing of either gene in immortalized Schwann cells and HEK293 cells had negligible effects on cellular proliferation in a Cell Titer-Blue viability assay (Supplementary Fig. 5c), their combined silencing resulted in decreased cellular viability (Supplementary Fig. 5d), suggesting that synthetic sickness might be the molecular basis for the mutual exclusivity of *ATP6AP1* and *ATP6AP2* mutations in GCTs. Moreover, treatment with *N*-ethylmaleimide (NEM; an H^+^-ATPase inhibitor that blocks vesicular transport) of immortalized Schwann cells and HEK293 cells, where ATP6AP1 or ATP6AP2 had been stably silenced, led to a significant decrease in cellular viability compared to controls, suggesting that the decreased endosomal acidification due to ATP6AP1 or ATP6AP2 loss of function might render cells susceptible to further V-ATPase inhibition (Supplementary Fig. 5e).

**Fig. 6:**
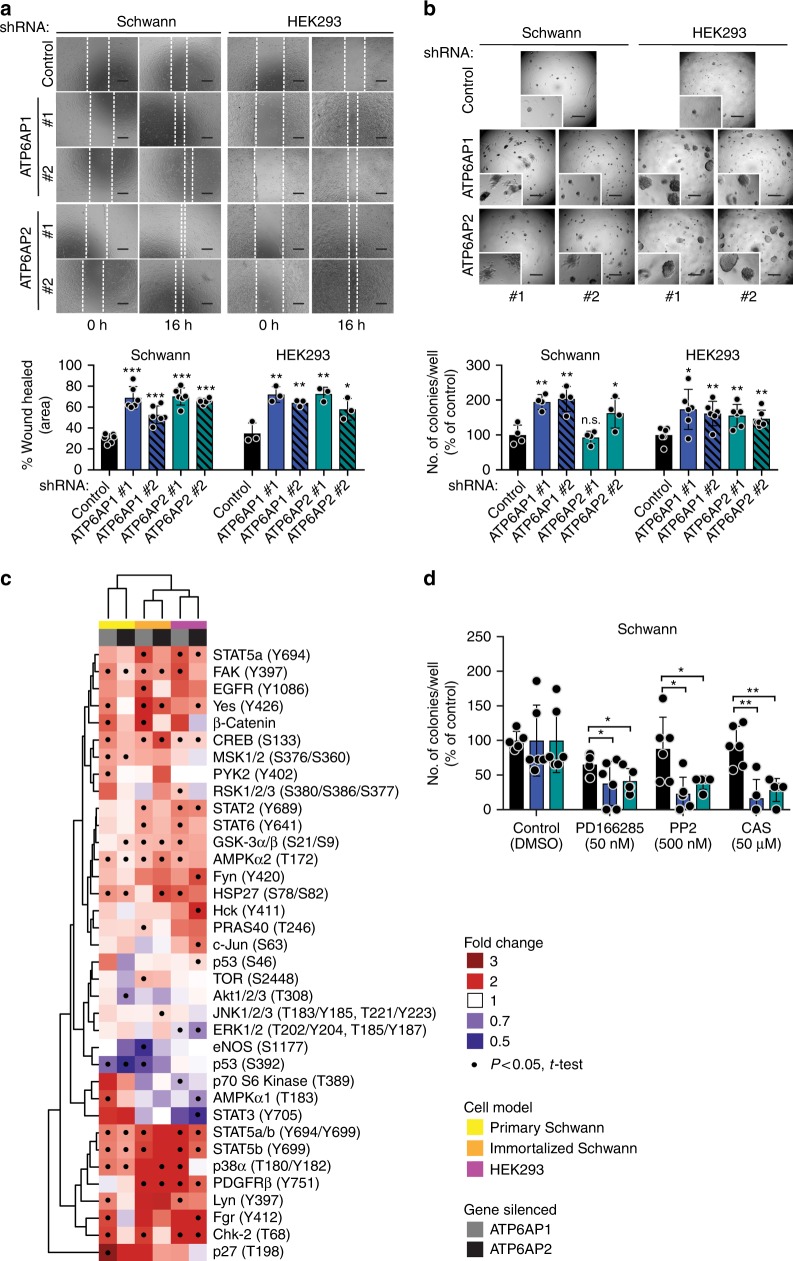
Loss of function of ATP6AP1 and ATP6AP2 confers oncogenic properties in vitro. **a** Wound healing assay of immortalized Schwann cells and HEK293 cells with stable silencing of ATP6AP1 or ATP6AP2 using short-hairpin RNAs (shRNAs). Wound area was assessed at 0 and 16 h. Scale bars, 500 μm. Quantification (bottom) of wound healed compared to control. Error bars, mean ± S.D. Schwann cells, *n* = 6; HEK293 cells, *n* = 3. *P < 0.05, **P < 0.01, ***P < 0.001; two-tailed unpaired *t*-test. **b** Soft agar colony formation assay of immortalized Schwann cells and HEK293 cells with stable shRNA silencing of ATP6AP1, ATP6AP2, or control shRNAs. Scale bars, 500 μm. Quantification (bottom) of number of colonies/well compared to control. Error bars, mean ± S.D. Schwann cells, *n* = 4; HEK293 cells, *n* = 6. n.s., non significant; *P < 0.05, **P < 0.01; two-tailed unpaired *t*-test. **c** Heatmap depicting phosphorylation levels of selected target proteins upon transient (primary Schwann) or stable (immortalized Schwann and HEK293 cells) RNA-interference silencing of ATP6AP1 or ATP6AP2, relative to control. Protein phosphorylation fold change, cell model, and gene silenced are color-coded according to the legend. Only proteins significantly (*P* < 0.05; two-tailed unpaired *t*-test) altered in at least one cell model are shown. Experiments were performed in replicates (primary Schwann cells, *n* = 4; immortalized Schwann cells, *n* = 4; HEK293 cells, *n* = 4). **d** Soft agar colony formation assay of immortalized Schwann cells with stable silencing of ATP6AP1, ATP6AP2, or control following 14 days of treatment with 50 nM of PD166285, 500 nM of PP2, and 50 μM of CAS 285986-31-4 (CAS) or vehicle (DMSO). Error bars, mean ± S.D. (*n* > 5) *P < 0.05, **P < 0.01; two-tailed unpaired *t*-test

We observed that stable silencing of ATP6AP1 or ATP6AP2 in immortalized Schwann cells and HEK293 cells, and transient silencing of either gene in primary Schwann cells, enhanced cellular migration in wound healing assays (Fig. 6a and Supplementary Fig. 5f), and resulted in an increased number of colonies and average colony size in a soft agar colony formation assay (Fig. 6b and Supplementary Fig. 5g). The increased cellular migration and anchorage-independent growth we observed upon silencing of ATP6AP1 or ATP6AP2 indicates that loss of function of either gene results in the acquisition of oncogenic properties in vitro, supporting a novel tumor suppressor role for *ATP6AP1* and *ATP6AP2* and the notion that inactivating mutations targeting these genes are potential drivers of GCTs.

Endocytosis orchestrates an array of cellular processes, including signaling, as it fine-tunes the amplitude and duration of various signaling cascades^13^. Hence, we posited that altered endocytosis downstream of ATP6AP1 or ATP6AP2 loss of function would result in the activation of signaling hubs. To identify the signaling cascades that may be triggered by ATP6AP1 and ATP6AP2 loss of function, we assessed the phosphorylation status of 43 kinases and 2 related proteins upon transient silencing of ATP6AP1 or ATP6AP2 in primary Schwann cells and stable silencing of either gene in HEK293 cells and immortalized Schwann cells. These analyses revealed that depletion of these genes resulted in increased phosphorylation of multiple signaling hubs, such as focal adhesion kinase (FAK) and its downstream targets p-38α and Hsp27^14^, Src-family kinases (SFKs) and signal transducer and activator of transcription 5a/b (STAT5a/b), 5'-adenosine monophosphate-activated protein kinase-α2 (AMPKα2) and its target CREB, which facilitates cancer cell adaptation to metabolic stress^15^, glycogen synthase kinase-3β (GSK3β), a key component of the Wnt pathway^16^, and platelet-derived growth factor receptor β (PDGFR-β) (Fig. 6c). To determine whether the oncogenic properties elicited by ATP6AP1 or ATP6AP2 loss of function may be attributed to increased signaling via the pathways above, we inhibited them pharmacologically, and assessed whether the oncogenic phenotype observed due to silencing of these genes would be reversed. Treatment with PD166285 (a protein tyrosine kinase inhibitor which potently targets PDGFR-β^17^), PP2 (a selective inhibitor of SFKs (which inhibits Lck, Fyn, and Hck robustly^18^), and the STAT5 inhibitor CAS 285986-31-4^19^ partially reversed the increased anchorage-independent growth elicited by stable silencing of ATP6AP1 or ATP6AP2 in immortalized Schwann cells (Fig. 6d). In addition, treatment with PD166285 and PP2 blocked the increased cellular migration observed upon depletion of ATP6AP1 or ATP6AP2 (Supplementary Fig. 5h). These results demonstrate that the oncogenic properties downstream of ATP6AP1 or ATP6AP2 loss of function might be mediated, at least to some extent, by increased signaling via PDGFR-β, SFKs, and STAT5 pathways.

## Discussion

Here, we demonstrate that GCTs harbor recurrent, mutually exclusive, and clonal somatic loss-of-function mutations affecting *ATP6AP1* and *ATP6AP2*. These genes encode for accessory proteins of the V-ATPase, which plays a pivotal role in the regulation of endosomal pH^7^. Missense germline mutations of *ATP6AP1* have been shown to be causative of Immunodeficiency 47^20^, characterized by hepatopathy, cognitive impairment, and abnormal protein glycosylation, whereas missense germline mutations affecting *ATP6AP2* result in the Hedera-type X-linked mental retardation syndrome^21,22^. Importantly, however, the role of loss-of-function germline mutations affecting *ATP6AP1* and *ATP6AP2* remains to be determined and neither *ATP6AP1* nor *ATP6AP2* have been previously implicated in cancer. We and others have shown that a subset of rare tumors arising in multiple anatomic sites are not uncommonly underpinned by recurrent, specific, or even pathognomonic, genetic alterations^23,24^. In this study, we expand the spectrum of rare cancer types underpinned by likely pathognomonic genetic alterations, as loss-of-function mutations targeting *ATP6AP1* or *ATP6AP2* are present in up to 72% of GCTs, but are found in less than 0.1% of common cancer types (Fig. 3a, b) and in none of the histologic mimics of GCTs tested here (Fig. 3c).

The histogenesis of GCTs remains a matter of contention. While it has been suggested that GCTs derive from Schwann cells^1^, other cell lineages have also been proposed^25–27^. Due to the lack of representative cell line models derived from human GCTs, we established cell models using Schwann cells, the likeliest cell of origin of GCTs, and HEK293 cells, where we stably silenced ATP6AP1 and ATP6AP2. The in vitro studies we conducted revealed that depletion of ATP6AP1 or ATP6AP2 results in accumulation of intracytoplasmic granules, recapitulating the cardinal histologic and ultra-structural features of human GCTs (Fig. 4a, b), likely due to accumulation of endosomal compartments with higher pH. These results support a genotypic–phenotypic correlation between loss-of-function mutations affecting *ATP6AP1* and *ATP6AP2* and GCTs. Interestingly, the accumulation of intracytoplasmic granules was more conspicuous in Schwann cells than in epithelial cells, suggesting that the interplay between loss of function of ATP6AP1 and ATP6AP2 and cell of origin is a likely determinant of the novel genotypic–phenotypic correlation described here.

We observed that loss of function of ATP6AP1 and ATP6AP2 results in decreased V-ATPase activity and endosomal acidification, likely due to reduced levels of the V0 domain of the V-ATPase, and decreased assembly of the V0 and V1 V-ATPase domains. The reduced levels of V-ATPase V0 domain are in agreement with Kinouchi et al.^28^, who reported on decreased V-ATPase V0 domain levels in floxed mouse embryonal fibroblasts following *Atp6ap2* deletion. Notably, in yeast, the loss of any V0 subunit may affect the stability of the remaining V0 subunits, and the assembly of the yeast V-ATPase requires assembly factors, such as *Vma12p*, *Vma21p*, and *Vma22p*^29^, and mutant cells lacking these factors display low levels of the V-ATPase V0 domain^30^. One could posit that ATP6AP1 and ATP6AP2 activity may be required for the assembly of the V-ATPase in humans, and that their loss of function results in decreased stability of the V0 domain with a subsequent reduction of V-ATPase functional levels.

Our functional studies demonstrated that ATP6AP1 and ATP6AP2 silencing results in the acquisition of oncogenic properties in vitro, which are partially dependent on signaling via PDGFR-β, SFKs, and STAT5. Endocytosis and cell signaling are intimately related cellular processes^31^, and it is therefore not surprising that loss of function of ATP6AP1 and ATP6AP2 leads to the activation of several signaling pathways. It should be noted, however, that defective endocytosis downstream of inactivation of either of these genes is possibly the common underlying mechanistic basis resulting in activation of different signaling pathways. Further studies are warranted to define the mechanistic links between ATP6AP1 and ATP6AP2 loss of function and altered signaling.

Our study has several limitations. Due to the multi-institutional nature of our study, follow-up information was unavailable, and an assessment of the impact of *ATP6AP1* and *ATP6AP2* mutational status on patient outcome could not be performed. It is possible that altered endocytosis with accumulation of endosomal organelles due to a deficient V-ATPase and the oncogenic phenotype downstream of overt signaling via PDGFR-β, SFKs, and STAT5 are phenomena due to loss of function of ATP6AP1 or ATP6AP2, but independent of each other.

In conclusion, *ATP6AP1* and *ATP6AP2* loss-of-function mutations are the likely drivers of GCTs, and appear to be pathognomonic for these tumors. Our findings provide a genotypic–phenotypic correlation and indicate that the intracytoplasmic granules of GCTs may constitute accumulated high-pH cytoplasmic vesicles resulting from defects in vesicle acidification. Finally, by studying a rare tumor type we have uncovered a novel potential tumor suppressor role for genes essential to endosomal pH control and vesicular trafficking.

## Methods

### Subjects and samples

Following approval by the IRB of  the authors’ institutions, frozen and/or formalin-fixed paraffin-embedded (FFPE) tissue blocks of GCTs were retrieved from the pathology archives/tissue banks of Memorial Sloan Kettering Cancer Center (MSKCC; NY, USA), Cleveland Clinic (OH, USA), University of Nottingham (UK), Thomas Jefferson University Hospital (PA, USA), and Edelweiss Laboratory (Brazil). Patient consents were obtained if required by IRB protocols approved by the authors’ institutions. Samples were anonymized before tissue processing. All cases were centrally reviewed by four pathologists (F.P., F.C.G., T.H., and M.E.) and assessed following the Fanburg-Smith criteria^32^. In total, after central pathology review, 82 tumors (5 frozen, 77 FFPE) arising from different anatomic locations, excluding the central nervous system, were classified by the four study pathologists as GCTs and included in this study.

The discovery series comprised 17 GCTs (Fig. 1, Supplementary Table 1). DNA was extracted separately from microdissected tumor and matched normal tissue samples under a stereomicroscope and subjected to WES. RNA was extracted from 11 microdissected GCTs, 10 of which were also subjected to WES, and subjected to RNA sequencing. The validation series consisted of 65 GCTs; FFPE-derived DNA from microdissected tumor and matched normal tissue samples was subjected to massively parallel sequencing using a custom bait set targeting all exons and flanking intronic regions of *ATP6AP1* and *ATP6AP2* (Fig. 1, Supplementary Table 1). In addition, DNA extracted from microdissected tumor and normal tissue samples from 103 GCT histologic mimics (Fig. 1) was subjected to *ATP6AP1* and *ATP6AP2* targeted massively parallel sequencing. For power calculations, we assumed that GCTs would be driven by a highly recurrent mutation or fusion gene present in ≥70% of cases, akin to other rare tumor types^33,34^. Based on a binomial distribution, the analysis of 10 cases would be sufficient to identify a highly recurrent genetic alteration or a highly recurrently altered gene with >90% statistical power.

### RNA sequencing and fusion gene identification

RNA sequencing was performed on 11 GCTs using validated protocols^23,35^ employed at MSKCC Integrated Genomics Operation (IGO). In brief, paired-end RNA sequencing was performed with 2 × 50 bp cycles on an Illumina HiSeq2000. Read pairs supporting fusion transcripts were identified using INTEGRATE^36^ and deFuse^37^, as previously described^23,35^. Candidates supported by at least two spanning reads were included. To account for alignment artifacts and normal transcriptional variants, we excluded fusion gene and read-through candidates which were identified in a set of 38 normal samples from TCGA^38^. The remaining candidate fusion genes were annotated using OncoFuse^39^ to define their likelihood of constituting potential driver fusion genes.

### Whole-exome sequencing and variant calling

Microdissected tumor and normal DNA samples from 17 GCTs were subjected to WES at MSKCC IGO using validated protocols as previously described^23,40^. Sequencing data were analyzed as previously described^40^. In brief, reads were aligned to the reference human genome GRCh37 using the Burrows-Wheeler Aligner (BWA, v0.7.10)^41^. Local realignment, duplicate removal, and base quality recalibration were performed using the Genome Analysis Toolkit (GATK, v3.1.1)^42^. Somatic single-nucleotide variants (SNVs) were detected by MuTect (v1.0)^43^, and small insertion and deletions (indels) by Strelka (v2.0.15)^44^ and VarScan2 (v2.3.7)^45^. SNVs and indels for which the tumor mutant allele fraction (MAF) was <5 times that of the matched normal MAF were excluded^46^. SNVs and indels found at >5% global minor allele frequency in dbSNP (build 137) were also excluded. All mutations were manually inspected using the Integrative Genomics Viewer (IGV)^47^. FACETS^48^ was used to determine CNAs and whether genes harboring a somatic mutation were targeted by loss of heterozygosity, as previously described^40,46^. ABSOLUTE (v1.0.6)^49^ was employed to determine the cancer cell fraction (CCF) of each mutation, as previously described^40,46^. A mutation was classified as clonal if its probability of being clonal was >50%^50^ or if the lower bound of the 95% confidence interval of its CCF was >90%^40,46^. Mutations affecting hotspot codons^51^ were annotated as previously described^46^.

### Validation of *ATP6AP1* and *ATP6AP2* mutations

DNA from GCTs of the validation series and DNA from histologic mimics of GCTs were subjected to targeted capture massively parallel sequencing using a custom bait set targeting all exons and flanking intronic regions of *ATP6AP1* and *ATP6AP2* (IDT Technologies). Sequencing read alignment and local realignment, duplicate removal, and quality score recalibration were performed using BWA^41^ and GATK^42^ as described above. SNVs were identified using MuTect^43^; indels were identified using Strelka^44^, VarScan2^45^, Platypus^52^, and Scalpel^53^, and further curated by manual inspection using IGV^47^.

All somatic *ATP6AP1* and *ATP6AP2* mutations identified by WES in GCTs from the discovery series were validated by Sanger sequencing, as previously described ^23^. In addition, all somatic *ATP6AP1* and *ATP6AP2* mutations identified by targeted sequencing in GCTs from the validation series were validated by Sanger sequencing ^23^ and/or by repeated targeted capture massively parallel sequencing using an independent DNA sample utilizing our custom *ATP6AP1* or *ATP6AP2* bait set (Supplementary Table1).

### Bisulfite sequencing and modified HUMARA assay

For bisulfite sequencing^54^ of *ATP6AP1* mutations near CpG islands, 200 ng of tumor DNA was treated with a bisulfite conversion kit (Methylamp DNA Modification Kit; EpiGentek), and amplified by PCR with methylated and non-methylated DNA-specific primers. For the modified HUMARA assay^55^ following methylation-specific DNA digestion^56^, 200 ng of tumor DNA was incubated overnight at 37 °C with 25 U of the methylation-sensitive restriction enzyme *Hha*I (New England Biolabs) or water (for mock-digested control). PCR fragments were cleaned using ExoSAP-IT (ThermoFisher Scientific) and subjected to Sanger sequencing as previously described^23^.

### Expression of mutant *ATP6AP1* and *ATP6AP2*

The mRNA expression of mutant *ATP6AP1* and *ATP6AP2* in GCTs identified by WES was assessed in the cases subjected to RNA sequencing by visual inspection of reads in IGV^47^. In addition, the expression of *ATP6AP1* and *ATP6AP2* mutations in GCTs identified by targeted sequencing was assessed by Sanger sequencing of RNA-derived cDNA. PCR fragments were cleaned using ExoSAP-IT (ThermoFisher Scientific) and Sanger sequenced as previously described^23^.

### Mutual exclusivity test

Mutual exclusivity analysis of mutations targeting *ATP6AP1* and *ATP6AP2* was performed using CoMEt^57^, a statistical approach to identify combinations of mutually exclusive alterations in cancer, as previously described^58^.

### Amino acid sequence alignment of ATP6AP1 across species

To determine whether ATP6AP1 residues affected by in-frame indels are evolutionarily conserved, the amino acid sequences of the ATP6AP1 protein from different species were retrieved from Ensembl and aligned using Clustal Omega at EMBL-EBI^59^, as previously described^23^.

### Analysis of data from TCGA

*ATP6AP1* and *ATP6AP2* mutation frequencies in 6285 common cancer (non-GCT) samples from TCGA were obtained from cBioPortal^6^, including 14 studies (see Supplementary methods). Following the approach described by Jelinic et al.^60^, *ATP6AP1* and *ATP6AP2* mutation frequencies were assessed following exclusion of hypermutated cases, defined as cancers harboring more than 1000 non-synonymous mutations, microsatellite unstable or harboring *POLE* or *POLD1* exonuclease domain mutations. Mutation diagrams (‘lollipop’ plots) were generated using MutationMapper on cBioportal^6^ and manually curated.

### Immunofluorescence

HEK293 (20,000 cells/well) and primary Schwann cells (7500 cells/well) were plated on Millicell EZ SLIDE 8-chamber slides, fixed with 4% formaldehyde (ThermoFisher Scientific), and stained using primary antibodies against EEA1 (Clone C45B10; Cell Signaling; #3288), Rab13 (Sigma-Aldrich; #SAB4200058), and LAMP1 (Clone D2D11; Cell Signaling; #9091S), followed by Alexa Fluor–conjugated secondary antibodies (ThermoFisher Scientific), at recommended dilutions. Slides were mounted using ProLong Gold Antifade Reagent with 4-6-diamidino-2-phenylindole (DAPI; ThermoFisher Scientific).

Formalin-fixed cell pellets were generated, as previously described^61^. Immunofluorescence staining of FFPE tumor and cell pellet sections was performed using the Leica Bond RX automated stainer (Leica Biosystems) using primary antibodies against ATP6AP1 (OriGene; #TA590072; 1:150) or ATP6AP2 (Sigma; #HPA003156; 1:200), followed by Alexa Fluor^TM^ 488 Tyramide Reagent (ThermoFisher Scientific; #B40953) and DAPI solution (ThermoFisher Scientific), according to the manufacturer’s instructions.

Fluorescence images were acquired with an Axio Imager 2 upright microscope (Zeiss) with a 40×/0.75 air objective, an Axiocam 506 camera (Zeiss), and Zen Blue (version 2) acquisition software (Zeiss) at a resolution of 1376 × 1104 pixels, a scaling of 0.227 micron/pixel, and a bit depth of 14-bit. DAPI and Alexa488 were illuminated with an Illuminator HXP 120 V(D) lamp (Zeiss) and imaged in the range of 435–485 nm and 512–542 nm, respectively. Linear LUT was used at full range. No post-acquisition processing was done, besides minor adjustments of brightness and contrast, applied equally to all images. ImageJ software was used to quantify the signal intensity per cell; at least five representative images (40× field) were analyzed for each case.

Confocal images were acquired with a TCS SP5 Upright Confocal microscope (Leica Microsystems), using a 63×/1.40 oil objective, HyD hybrid detectors, and the Leica Application Suite Advanced Fluorescence (LASAF) acquisition software (Leica Microsystems), as previously described^62^. Images were acquired at a resolution of 1024 × 1024 pixels, a scaling of 0.12 micron/pixel, and an 8-bit depth. DAPI, Alexa568 and Alexa488 were excited with 405 nm, 543 nm, and 488 nm lasers, respectively, and imaged in the range of 410–460 nm, 570–620 nm, and 490–520 nm, respectively. Linear LUT was used at full range. No post-acquisition processing was performed, besides minor adjustments of brightness and contrast, applied equally to all images. ImageJ was used to quantify the number of foci per cell, with at least 7 representative images analyzed for each condition. All experiments were repeated three times.

### Cell lines

HEK293 (ATCC), MCF-10A (ATCC), primary Schwann (ScienCell), and immortalized human normal Schwann cells (Margaret Wallace, University of Florida)^63^ were authenticated using short tandem repeat profiling at MSKCC IGO and tested for mycoplasma using the PCR-based Universal Mycoplasma Detection kit (ATCC). HEK293 and immortalized Schwann cells were cultured in Dulbecco's modified Eagle's medium (DMEM) high glucose supplemented with 10% fetal bovine serum (FBS) and 1% penicillin/streptomycin. MCF-10A cells were cultured in DMEM/F12 supplemented with 5% horse serum, 20 ng/ml epidermal growth factor, 10 μg/ml insulin, 0.5 μg/ml hydrocortisone, and 1% penicillin/streptomycin. Primary Schwann cells were cultured in Schwann Cell Medium (ScienCell), consisting of basal medium, 5% FBS, 1% Schwann cell growth supplement, and 1% penicillin/streptomycin. All cell lines were maintained in a 5% CO_2_ atmosphere at 37 °C.

### Inhibitors

The ATPase inhibitor NEM (#34115, Millipore Sigma) was used in cell viability assays. The V-ATPase inhibitor Bafilomycin-A1 (Millipore Sigma; #B1793) was used in LC3B western blots. PP2 (R&D Systems; #1407), an inhibitor of the Src family kinases, PD166285 (#3785, R&D Systems), a Src, PDGFRβ and FGFR1 inhibitor, and CAS 285986-31-4 (Millipore Sigma; #573108), a STAT5 inhibitor, were used in colony formation and wound healing assays (see below).

### Transient siRNA transfection

Transient transfections were performed using Lipofectamine RNAiMAX (ThermoFisher Scientific) with ON-TARGETplus SMART pool short-interfering RNA (siRNA) for human ATP6AP1 and ATP6AP2, single ON-TARGETplus siRNAs for human ATP6AP1 and ATP6AP2, and ON-TARGETplus Non-Targeting control pool (GE Healthcare Dharmacon), according to the manufacturer’s instructions. Transfection efficiency was assessed by western blotting (see below) and by TaqMan quantitative reverse transcription-polymerase chain reaction (qRT-PCR); ATP6AP1 (Hs00184593_m1) and ATP6AP2 (Hs00997145_m1) gene expression levels were evaluated, using GAPDH (Hs02786624) for normalization, as previously described^64^. Experiments were performed 72 h after transfection and repeated a minimum of three times for each condition.

### Generation of stable cell lines

Custom high efficiency miR-E short-hairpin RNAs (shRNAs) in the SGEP vector (puromycin/GFP) targeting ATP6AP1 or ATP6AP2 were created by the MSKCC RNAi Core Facility, as previously described^65^. miR-E shRNA targeted to Firefly *Renilla* Luciferase was used as a negative, non-targeting control. Virus containing shRNA was produced in HEK293T cells (ATCC) using a lentiviral packaging mix (Sigma-Aldrich). In addition, Mission shRNA lentiviral transduction particles (Sigma-Aldrich) targeting ATP6AP1 or ATP6AP2 or non-targeting control were employed. HEK293 or immortalized Schwann cells were infected for 48 h with virus containing shRNA and then selected for 14 days in puromycin (2 µg/ml;ThermoFisher Scientific). Silencing efficiency was confirmed by qRT-PCR and western blot, and for ATP6AP1 or ATP6AP2, the two shRNAs with the highest level of silencing were selected for downstream experiments.

### Transmission electron microscopy

Cells were fixed with a modified Karnovsky’s fixative, and embedded in an epon analog resin. En face ultrathin sections (65 nm) were contrasted with lead citrate, as previously described^66^. FFPE tissue punches were reprocessed and embedded in flat molds and processed following the above protocol^66^.

Transmission electron micrographs were captured using a JSM 1400 electron microscope (JEOL), a Veleta 2K x 2K CCD camera (EMSIS), and the iTEM acquisition software (EMSIS). Images were acquired at a resolution of 4008 × 2672 pixels at 9 micron/pixel and a 14-bit depth. Linear LUT was used at full range. No post-acquisition processing was performed, besides minor adjustments of brightness and contrast, applied equally to all images. At least 10 representative images (8000× field) were analyzed per condition using ImageJ.

### Protein blotting

Standard western blotting was conducted as previously described^67^. Frozen tissue was lysed in RIPA buffer and homogenized using a Tissuelyser (Qiagen). Proteins were extracted from FFPE material using the Qproteome FFPE Tissue Kit (Qiagen; #37623), according to the manufacturer’s instructions. Cell fractionation was performed using the Cell Fractionation Kit (Cell Signaling; #9038), according to the manufacturer’s instructions. Primary antibodies against ATP6AP1 (cell lines and cell pellets: Santa Cruz; #sc-81886; GCTs: Sigma-Aldrich; #A1486), ATP6AP2 (Sigma-Aldrich; #HPA003156), Tubulin (Cell Signaling; #2125), GAPDH (Clone 14C10; Cell Signaling; #2118), ATP6V1A (Abcam; #ab137574), ATP6V0D1 (Abcam; #ab56441), MEK1/2 (Clone D1A5; Cell Signaling; #8727), NRG1 (Abcam; #ab180808), LC3B (Cell Signaling; #2275), EEA1 (Clone C45B10; Cell Signaling; #3288), Rab13 (Sigma-Aldrich; #SAB4200058), and LAMP1 (Clone D2D11; Cell Signaling; #9091S) were used at recommended dilutions. Conjugated IRDye680RD/800CW secondary antibodies were employed and detected using the Odyssey Infrared Imaging System (LI-COR Biosciences). Quantification and analysis were performed using LI-COR Image Studio Software. Experiments were repeated in triplicate. Unprocessed images of all western blots are shown in Supplementary Figs. 6 and 7.

### Live-cell microscopy

HEK293 (20,000 cells/well) and primary and immortalized Schwann cells (7500 cells/well) were seeded on top of glass slides with polystyrene chambers (Lab-Tek II Chambered Coverglass slides; Nunc). Cells were incubated for 20 min at 37 °C with pHrodo Red dextran 10,000 MW probes (ThermoFisher Scientific; #P10361), at a concentration of 40 μg/ml, following the manufacturer’s guidelines. Confocal fluorescence and bright-field *z*-stacks were acquired at 37 °C using an LSM880 inverted confocal microscope (Zeiss) with a 25× objective, Gallium arsenide phosphide (GaAsP) array detectors, and ZEN Black (Version 2.3) acquisition software (Zeiss). Images were acquired at a resolution of 1024 × 1024 pixels, a scaling of 0.21 × 0.21 × 0.6 micron/pixel, and an 8-bit depth. pHrodo Red dextran was excited with a 561 nm laser and imaged in the range of 562–633 nm. Linear LUT was used at full range. No post-acquisition processing was performed, besides minor adjustments of brightness and contrast, applied equally to all images. Sum-intensity projection images were generated and total pHrodo Red dextran intensity per cell-covered area was quantified using ImageJ. Experiments were performed in triplicate.

### Flow cytometry

For endocytosis experiments, cells were incubated with 25 µg/ml pHrodo Red Transferrin conjugate (ThermoFisher Scientific; #P35376) at 37 °C for 20 min. Fluorescence was evaluated by flow cytometry using an LSR Fortessa (BD Biosciences) instrument with a 561 nm laser and a 582/15 nm BP for excitation and detection of pHrodo, respectively. The cell population was first gated in a FSC-A vs. SSC-A plot, followed by doublet discrimination using FSC-H vs. FSC-W. For experiments with GFP+ cells, the GFP+ population was gated using a 2D plot of 582/15 nm (561 nm excitation) vs. 530/50 nm (488 nm excitation). In all samples, a histogram of pHrodo fluorescence was measured from the final gated population (singlets or GFP+; Supplementary Fig. 8a).

For lysosomal activity experiments, the Lysosomal Intracellular Activity Assay Kit (Biovision; #K448) was used according to the manufacturer’s instructions. Cells were incubated with the lysosome-specific self-quenched substrate at 37 °C for 1 h. The lysosome-specific self-quenched substrate fluorescent signal was detected by flow cytometry using a 3-laser (405 nm, 488 nm; 640 nm) Aurora (Cytek Biosciences) spectral analyzer with 38 fluorescent channels and 2 scatter detectors (FSC and SSC). Raw spectral data were acquired and unmixed using Spectroflo Software (Cytek Biosciences) with autofluorescence extraction. The cell population was first gated in a FSC-A (488 nm) vs. SSC-A (405 nm) plot, followed by doublet discrimination using FSC-H vs. FSC-W (Supplementary Fig. 8b). Experiments were performed in triplicate and 10,000 events were acquired per sample. The MFI of each sample was evaluated using FCS express 6.04 (De Novo Software).

### V-ATPase activity assay

Total ATPase activity was measured in cell membrane protein using the ATPase/GTPase Activity Assay Kit (Sigma-Alrich; #MAK-113), according to the manufacturer’s instructions, in either the presence or absence of 250 nM Bafilomycin-A1. Absorbance at 595 nm was detected using a Victor X4 Multimode Plate Reader (PerkinElmer). The Bafilomycin-A1-sensitive ATPase activity was taken as a measure of V-ATPase activity. Experiments were performed in triplicate.

### Scratch wound healing assay

Cells were serum-starved and seeded in 24-well plates at 90–95% confluence in the CytoSelect 24-well Wound Healing Assay(Cell Biolabs, Inc), according to the manufacturer’s guidelines. Phase-contrast images were obtained at 0 h and 16 h following scratch wounding, using an EVOS XL Core Microscope (ThermoFisher Scientific) and analyzed using ImageJ. Experiments were performed in triplicate at least 3 times.

### Colony formation assay

Soft agar colony formation assay was performed using CytoSelect 96-well Cell Transformation Assay (Cell Biolabs, Inc.), according to the manufacturer’s guidelines. Briefly, cells were seeded in 1.2% agar in 96-well plates (1000 cells/well); *n* = 4 for immortalized Schwann cells and *n* = 6 for HEK293 cells). After 14 days, phase-contrast images were obtained using the EVOS XL Core Microscope (ThermoFisher Scientific). Quantification of the number of colonies per well and colony size was performed using ImageJ as previously described^23^.

### Proliferation assay

Cells were seeded in 96-well plates (1000 cells/well; *n* = 3 for immortalized Schwann cells and *n* = 4 for HEK293 cells). Proliferation rate was assessed using the Cell Titer-Blue Cell Viability Assay (Promega). Absorbance detection was performed with 560 nm excitation and 590 nm emission using a Victor X4 Multimode Plate Reader (PerkinElmer), as previously described. Experiments were performed in triplicate.

### Human phospho-kinase array

Relative phosphorylation levels of 43 kinases and 2 related proteins were assessed using the Proteome Profiler Human Phospho-Kinase Array Kit (R&D Systems), according to the manufacturer’s instructions. In brief, cell lysates were incubated overnight with nitrocellulose membranes of the Human Phospho-Kinase Array (R&D Systems). Membranes were then washed, incubated with biotinylated detection antibody cocktails, and then incubated with streptavidin-horseradish peroxidase and visualized using chemoluminescent reagents and X-ray film (GE Healthcare). Experiments were performed in four biological replicates. The signal of each capture spot was measured using the ‘Protein Array Analyzer for ImageJ’ and normalized to internal reference controls. The median value of the four replicates was calculated for each target, and the fold change compared to the control condition represented. Heteroscedastic Student’s *t*-test was used to determine statistical significance.

### Statistical analysis

Statistical analysis was performed using Prism 7 (GraphPad). A *P* value of <0.05 was considered significant. Student’s *t*-test was employed for the comparison of means in parametric data. The heteroscedasticity was assessed for each comparison, and homoscedastic or heteroscedastic *t*-tests were employed as appropriate. All *P* values were two-sided. The 95% confidence intervals were adopted.

## Electronic supplementary material

Supplementary Information

## Data Availability

Whole-exome sequencing and RNA sequencing data that support the findings of this study have been deposited in Sequence Read Archive with the accession codes SRP119539 and SRP118840 respectively.
