# Supplementary material for: Loss-of-function mutations in ATP6AP1 and ATP6AP2 in granular cell tumors
Source: Nat Commun. 2018 Aug 30;9:3533. doi: 10.1038/s41467-018-05886-y (PMC6117336; doi:10.1038/s41467-018-05886-y)
Supplement: Supplementary file 1 — Supplementary Information [file 41467_2018_5886_MOESM1_ESM.pdf]

Supplementary Materials

Loss-of-function mutations in *ATP6AP1* and *ATP6AP2* in granular cell tumors

Pareja et al.

Supplementary Methods

Supplementary Figures 1-8

Supplementary Tables 1-5

Supplementary References

SUPPLEMENTARY METHODS

The Cancer Genome Atlas (TCGA) studies re-analyzed in this study

For the analysis of the prevalence of *ATP6AP1* and *ATP6AP2* somatic mutations, whole-exome sequencing results pertaining to 6,285 non-hypermutated common cancer samples were retrieved from TCGA studies of 14 common cancer types, including Acute Myeloid Leukemia[1], Bladder Urothelial Carcinoma[2], Breast Invasive Carcinoma[3], Colorectal Adenocarcinoma[4], Head and Neck Squamous Cell Carcinoma[5], Kidney Renal Clear Cell Carcinoma[6], Kidney Chromophobe[7], Low-Grade Glioma/Glioblastoma Multiforme[8], Pan-Lung Cancer[9], Ovarian Serous Cystadenocarcinoma[10], Prostate Adenocarcinoma[11], Stomach Adenocarcinoma[12], Uterine Corpus Endometrial Carcinoma[13] and Papillary Thyroid Carcinoma[14].

Supplementary Figure 1

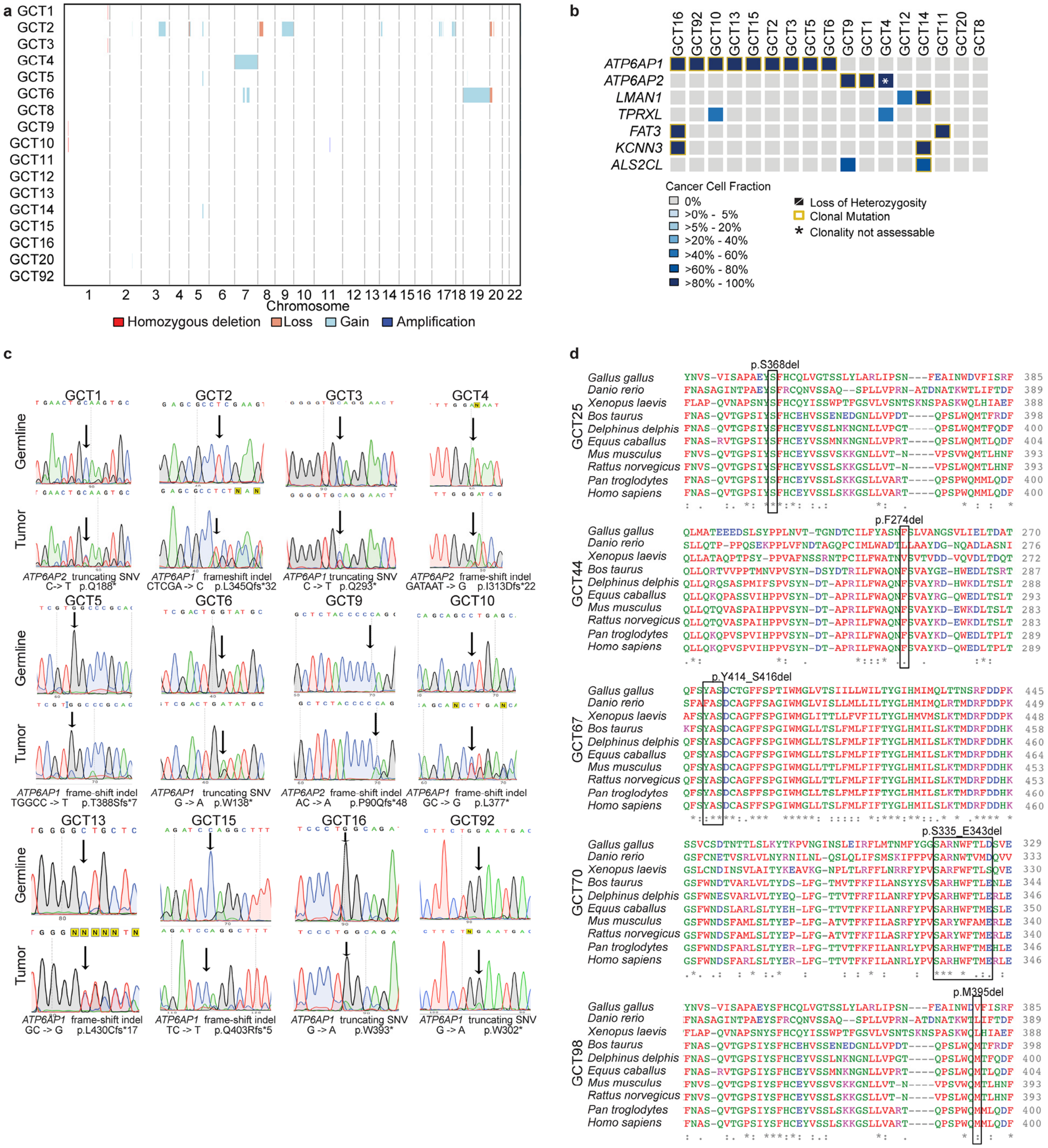

Supplementary Figure 1: Copy number profiles and clonality of recurrent non-synonymous mutations identified in granular cell tumors by whole-exome sequencing, Sanger sequencing validation of *ATP6AP1* and *ATP6AP2* mutations and evolutionary conservation of *ATP6AP1* in-frame indels.

(a) Copy number profiles of granular cell tumors (GCTs) subjected to whole-exome sequencing (WES; n=17). Samples are shown in rows, chromosomes are depicted along the x-axis, and copy number alterations are color-coded according to the legend. **(b)** Cancer cell fraction (CCF) and clonality of each recurrent non-synonymous somatic mutation identified in GCTs by WES (n=17). CCFs are color-coded according to the legend. Clonal mutations are depicted by an orange box. *, sequencing depth not appropriate for clonality assessment. **(c)** Representative sequence

electropherograms of normal and tumor samples from GCTs found to harbor *ATP6AP1* or *ATP6AP2* mutations by WES (n=12). The gene affected, mutation type, base and amino acid change are indicated for each case. Arrows point to the altered base. **(d)** Multiple alignment of the amino acid sequence of human ATP6AP1 with the orthologous proteins of *Gallus gallus, Danio rerio, Xenopus laevis, Bos taurus, Delphinus delphis, Equus caballus, Mus musculus, Rattus norvegicus* and *Pan troglodytes* for GCTs harboring *ATP6AP1* in-frame small insertion and deletion (indel) mutations (n=5). The residues altered by in-frame indels are indicated by black boxes. "*", single fully conserved residue; ":", conservation between groups of strongly similar properties; ".", conservation between groups of weakly similar properties. The physicochemical properties of the residues are color-coded as follows: small (red), acidic (blue), basic (magenta), and hydroxyl + sulfhydril + amine + G (green).

Supplementary Figure 2

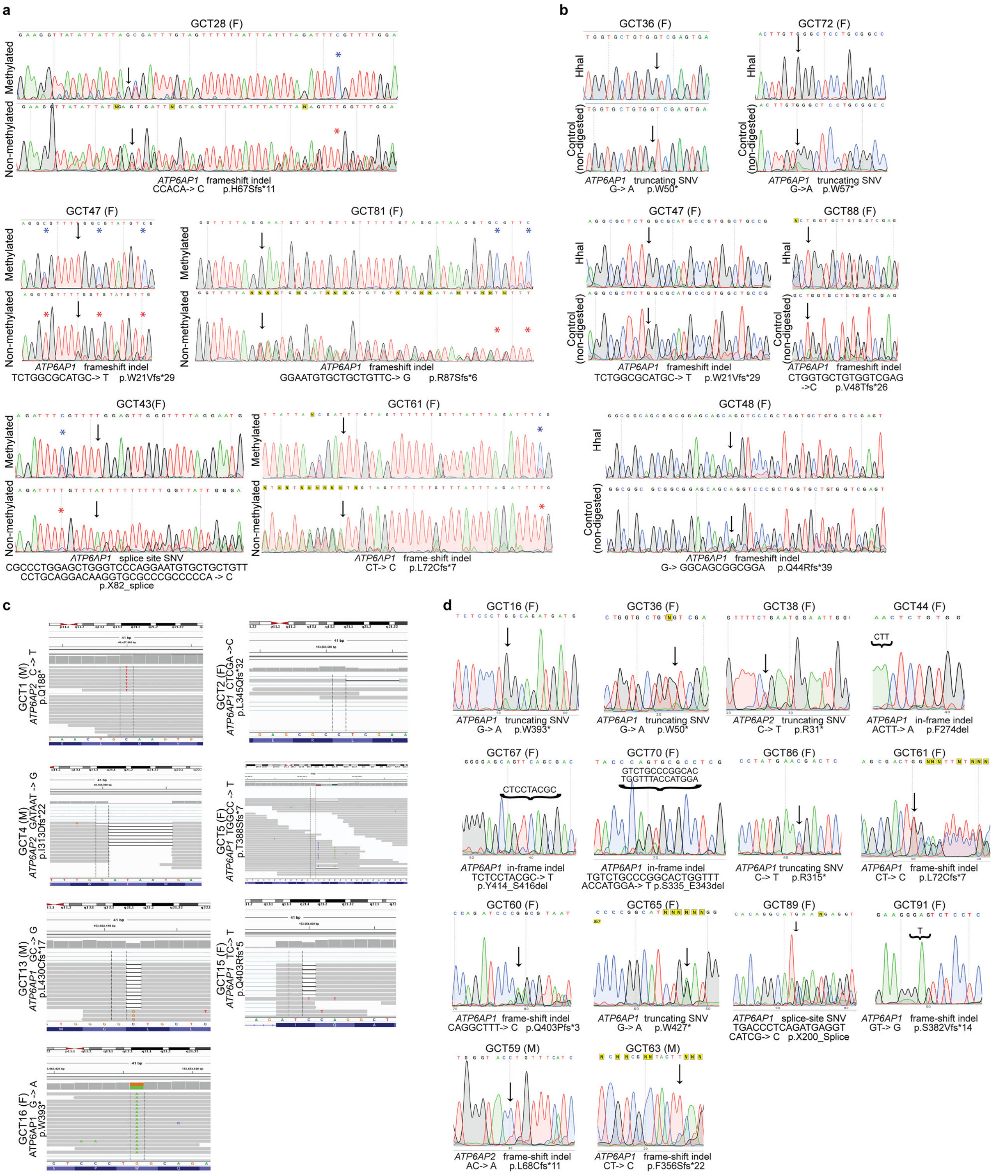

Supplementary Figure 2: *ATP6AP1* loss-of-function mutations and X chromosome inactivation, and expression of mutant *ATP6AP1* and *ATP6AP2* transcripts in granular cell tumors (GCTs).

(a) Representative bisulfite sequencing electropherograms of GCTs from female patients harboring *ATP6AP1* mutations in the vicinity of CpG islands (n=5). Following bisulfite treatment, DNA was PCR amplified with primer sets specific for methylated DNA (upper) and non-methylated DNA (lower). The gene affected, mutation type, base and amino acid change are indicated in each case. Arrows depict the altered base. Blue (*), methylated non-converted cytosine residues, red (*), non-methylated cytosine residues converted to thymidine. **(b)** Modified HUMARA assay representative sequence electropherograms of GCTs from female patients harboring *ATP6AP1* mutations in the vicinity of CpG islands (n=3), following restriction digestion with HhaI (upper) or control mock-digestion (lower). The gene affected, mutation type, base and amino acid change are indicated in each case. Arrows depict the altered base. **(c)** Integrative Genomics Viewer (IGV) pileups of the mRNA expression of ATP6AP1 and ATP6AP2 mutated forms identified by RNA-sequencing in GCTs from male (n=3) and female (n=4) patients. The gender, gene affected, base and amino acid changes are indicated for each case. **(d)** Representative sequence electropherograms of cDNA derived from RNA extracted from GCTs found to harbor *ATP6AP1* or *ATP6AP2* mutations by whole-exome sequencing (n=1) or targeted capture sequencing (n=13), including male (n=2) and female (n=12) patients. The gene affected, mutation type, base and amino acid change are indicated in each case. Arrows and brackets point to the altered base. F, female; M, male.

Supplementary Figure 3

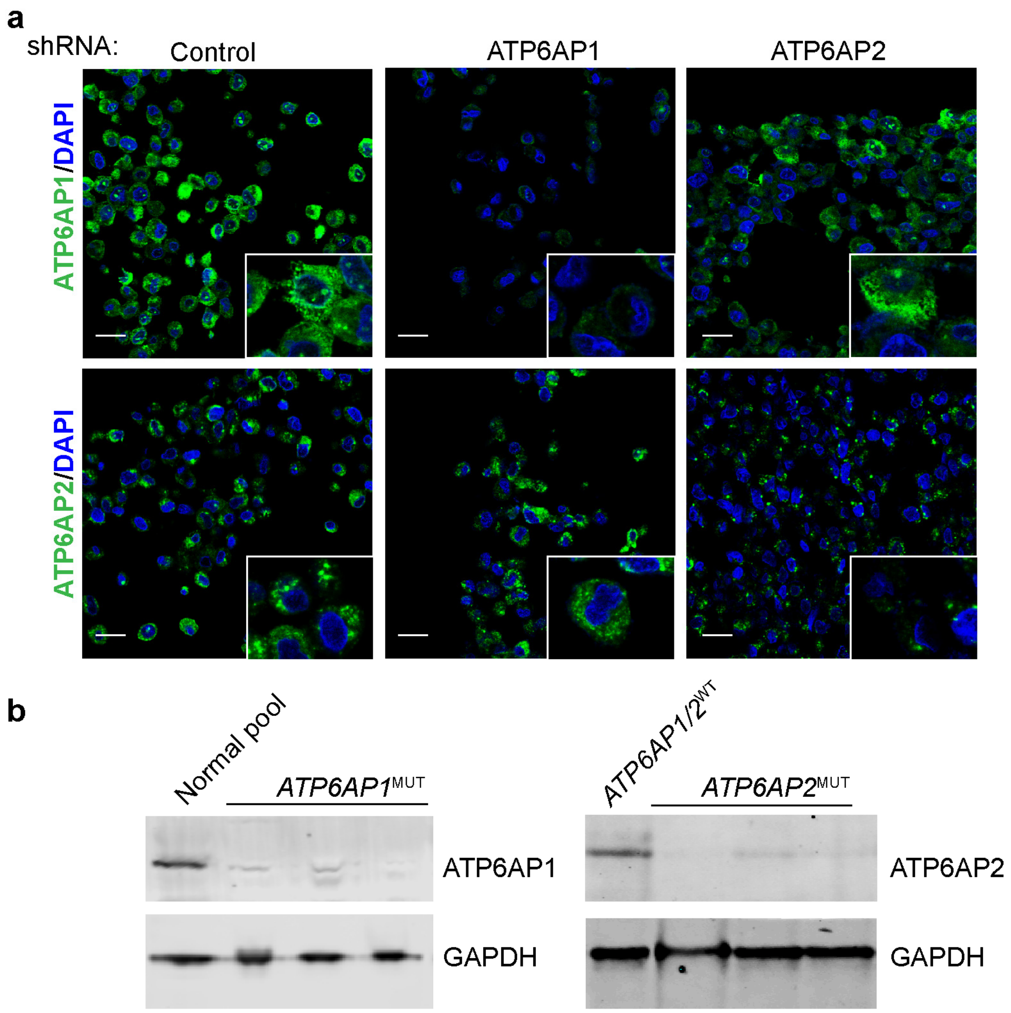

Supplementary Figure 3: *ATP6AP1* and *ATP6AP2* silencing and loss of function mutations result in loss of protein expression in granular cell tumors.
(a) Confocal fluorescence micrographs of formalin-fixed paraffin-embedded (FFPE) immortalized Schwann cell pellets where ATP6AP1 or ATP6AP2 had been stably silenced, or control cells. Scale bars, 25 µm. **(b)** Western blot analysis of ATP6AP1 and ATP6AP2 in human granular cell tumors (GCTs). Protein extracts from frozen *ATP6AP1*^{MUT} GCTs (n=3) and a pool of matching normal tissue were blotted for ATP6AP1. Protein extracts from FFPE *ATP6AP2*^{MUT} (n=3) and *ATP6AP1/2*^{WT} (n=1) GCTs were blotted for ATP6AP2. GAPDH was used as loading control. Experiments are representative of three replicates.

Supplementary Figure 4

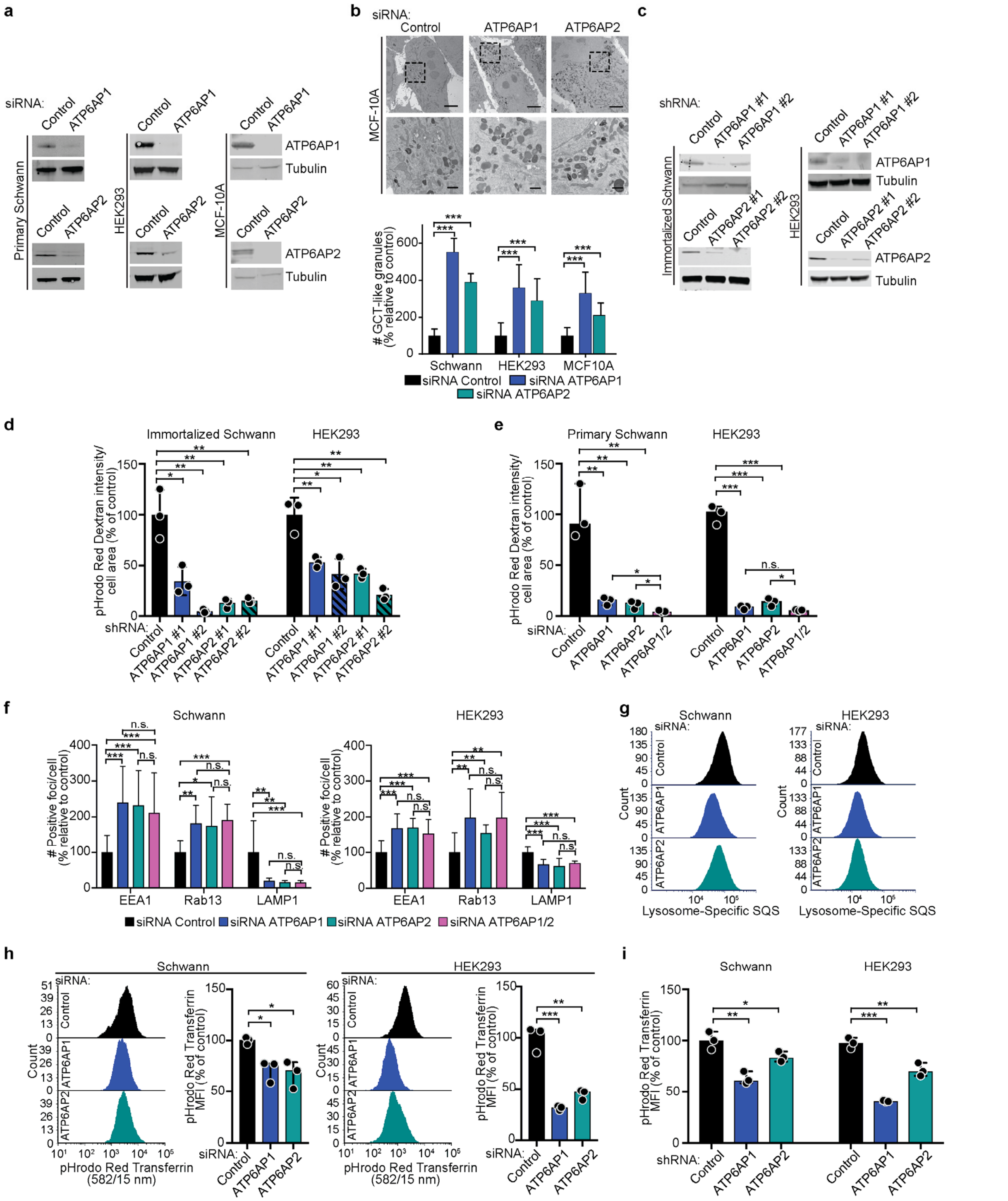

Supplementary Figure 4: Functional impact of ATP6AP1 or ATP6AP2 silencing on the phenotype, distribution of endosomal compartments, lysosomal acidification and activity, and endocytic flux.

(a) ATP6AP1 and ATP6AP2 western blot analysis of primary Schwann cells, HEK293 cells and MCF10A cells following transfection with validated short-interfering RNAs (siRNAs) targeting ATP6AP1 or ATP6AP2, or non-targeting control siRNA pools. Tubulin was used as loading control.

(b) Transmission-electron micrographs of MCF10A cells transfected with siRNAs targeting ATP6AP1 or ATP6AP2, or with non-targeting control siRNAs (top). Scale bars, 5 μm and 1 μm. Quantification of the number of GCT-like intracytoplasmic granules per image area of primary Schwann (from **Fig. 4b**), HEK293 (from **Fig. 4b**) and MCF10A cells following transfection with siRNAs targeting ATP6AP1 or ATP6AP2, or non-targeting control siRNAs (bottom). Quantification was performed using ImageJ in at least 10 representative images per condition (error bars, mean ± SD). **(c)** ATP6AP1 and ATP6AP2 western blot analysis of immortalized Schwann and HEK293 cells with stable short-hairpin (sh)RNA silencing of ATP6AP1 or ATP6AP2, or control shRNAs. Tubulin was used as loading control. **(d)** Quantification of pHrodo Red dextran fluorescence intensity per cell-covered area in immortalized Schwann cells and HEK293 cells with stable silencing of ATP6AP1 or ATP6AP2, using 2 different shRNA constructs per gene, or control non-targeting shRNA species (n=3, mean ± SD). Quantification was performed using ImageJ in at least 3 representative images per sample. **(e)** Quantification of pHrodo Red dextran fluorescence intensity per cell-covered area in primary Schwann and HEK293 cells following transient silencing of ATP6AP1 and ATP6AP2, singly or in combination, or non-targeting control (n=3, mean ± SD). Quantification was performed using ImageJ in at least 5 representative images per sample. **(f)** Quantification of the number of EEA1-positive (early endosomes), Rab13-positive (recycling endosomes) and LAMP-1 positive (lysosomes) foci in primary Schwann and HEK293 cells following transient silencing of ATP6AP1 and ATP6AP2, singly or in combination, or with non-targeting control siRNAs (n≥7, mean ± SD). The average number of foci per cell (DAPI stained nuclei) was analyzed using ImageJ in at least 10 representative images per condition. **(g)** Lysosomal activity assay in immortalized Schwann cells and HEK293 cells transfected with siRNAs against ATP6AP1 or ATP6AP2, or non-targeting control siRNAs. Flow cytometry histograms of immortalized Schwann cells and HEK293 cells incubated with Lysosomal-Specific Self-Quenched Substrate (SQS; n=3). **(h)** Flow cytometry histograms of primary Schwann cells and HEK293 cells incubated with pHrodo Red Transferrin conjugate. Quantification of the median fluorescence intensity (MFI) of the pHrodo Red signal (n=3; mean ± SD). **(i)** Quantification of the MFI of the pHrodo Red signal in immortalized Schwann cells and HEK293 cells with stable silencing of ATP6AP1 or ATP6AP2 and non-targeting shRNA control following incubation with pHrodo Red Transferrin conjugate and flow cytometry analysis (n=3; mean ± SD). P-values were determined using Student's t-test (***=p≤0.001, **=p≤0.01, *=p≤0.05, n.s.=p>0.05).

Supplementary Figure 5

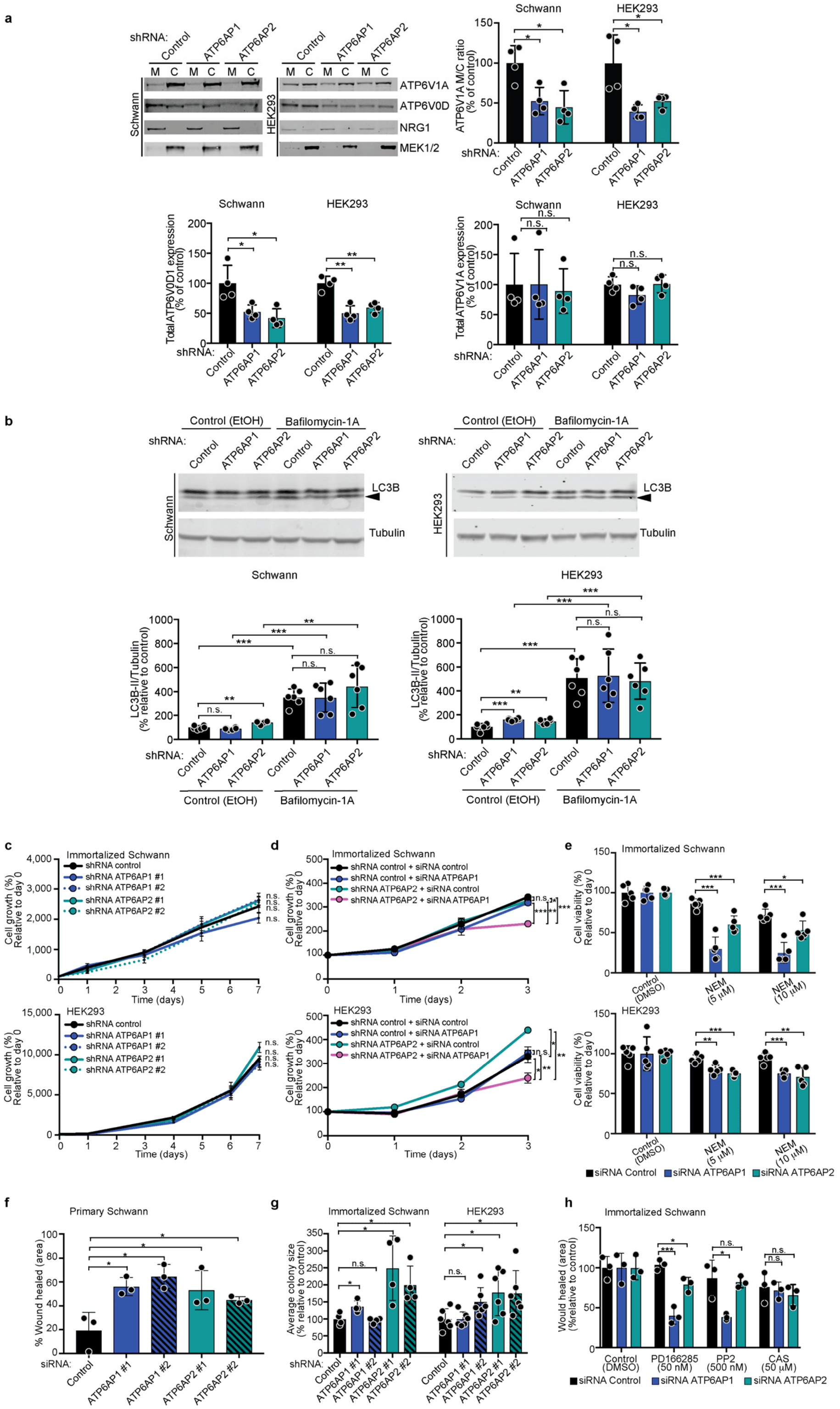

Supplementary Figure 5: Functional impact of ATP6AP1 or ATP6AP2 silencing on the assembly of the V-ATPase complex, autophagy, cell viability, cellular migration and anchorage independent growth, and susceptibility to pharmacologic inhibition.

(a) Western blot analysis of ATP6V1A and ATP6V0D expression in the membrane (M) and cytosolic (C) fractions of immortalized Schwann cells and HEK293 cells with stable short-hairpin (sh)RNA

silencing of ATP6AP1 or ATP6AP2, or non-targeting control shRNA. MEK1/2 and NRG1 were used as markers of the cytosolic and membrane fractions, respectively. Quantification of ATP6V1A membrane to cytosolic ratio (top), a surrogate of V-ATPase assembly, and total ATP6V0D1 and ATP6V1A expression (bottom) are shown (n=6; mean ± SD). **(b)** Western blot analysis of LC3B in immortalized Schwann cells and HEK293 cells at baseline conditions and following treatment with the Bafilomycin-1A. Whole cell lysates from immortalized Schwann cells (left) and HEK293 cells (right) with stable silencing of ATP6AP1 or ATP6AP2, and non-targeting controls were immunoblotted for LC3B following autophagy induction with Bafilomycin-1A or EtOH (vehicle control). Quantification LC3B-II (arrowheads)/tubulin ratios is depicted relative to control cells (Immortalized Schwann cells, n=5; HEK293 cells, n=4). **(c)** Cell proliferation assay of immortalized Schwann cells and HEK293 cells with stable ATP6AP1 or ATP6AP2 silencing over a period of 7 days (n=3, mean ± SD). **(d)** Cell proliferation assay of immortalized Schwann cells and HEK293 cells with single silencing of ATP6AP1 short-interfering RNA (siRNA, ATP6AP1) or ATP6AP2 (shRNA ATP6AP2), combined silencing of ATP6AP1 and ATP6AP2 (siRNA ATP6AP1 + shRNA ATP6AP2) or control (shRNA Control + siRNA Control) over a period of 3 days (n=3, mean ± SD). **(e)** Cell viability assay of immortalized Schwann cells and HEK293 cells with stable silencing of ATP6AP1 or ATP6AP2 with validated shRNAs, or control shRNA following treatment with different concentrations of N-ethylmaleimide (NEM) or control (DMSO) for 72 hours (n=5; mean ± SD). **(f)** Wound healing assay of primary Schwann cells following transfection with 2 individual siRNA oligonucleotides targeting ATP6AP1 or ATP6AP2 or non-targeting control siRNAs (n=3; mean ± SD). Wound area was quantified using ImageJ at 0 hours and 16 hours, and the percent of wound healed after 16 hours was determined (mean ± SD). **(g)** Soft agar colony formation assay of immortalized Schwann cells and HEK293 cells with stable silencing of ATP6AP1, ATP6AP2 or non-targeting control (Schwann cells, n=4; HEK293 cells, n=6; mean ± SD). Quantification of average colony size is shown. **(h)** Wound healing assay of immortalized Schwann cells with stable silencing of ATP6AP1, ATP6AP2 or non-targeting control, following treatment with the indicated concentrations of PD166285, PP2, CAS285986-31-4 or vehicle (DMSO). Quantification of the percent of wound healed after 16 hours is shown (n=3; mean ± SD). P-values were determined using Student's t-test (***=p≤0.001, **=p≤0.01, *=p≤0.05, n.s.=p>0.05). CAS, CAS285986-31-4.

Supplementary Figure 6

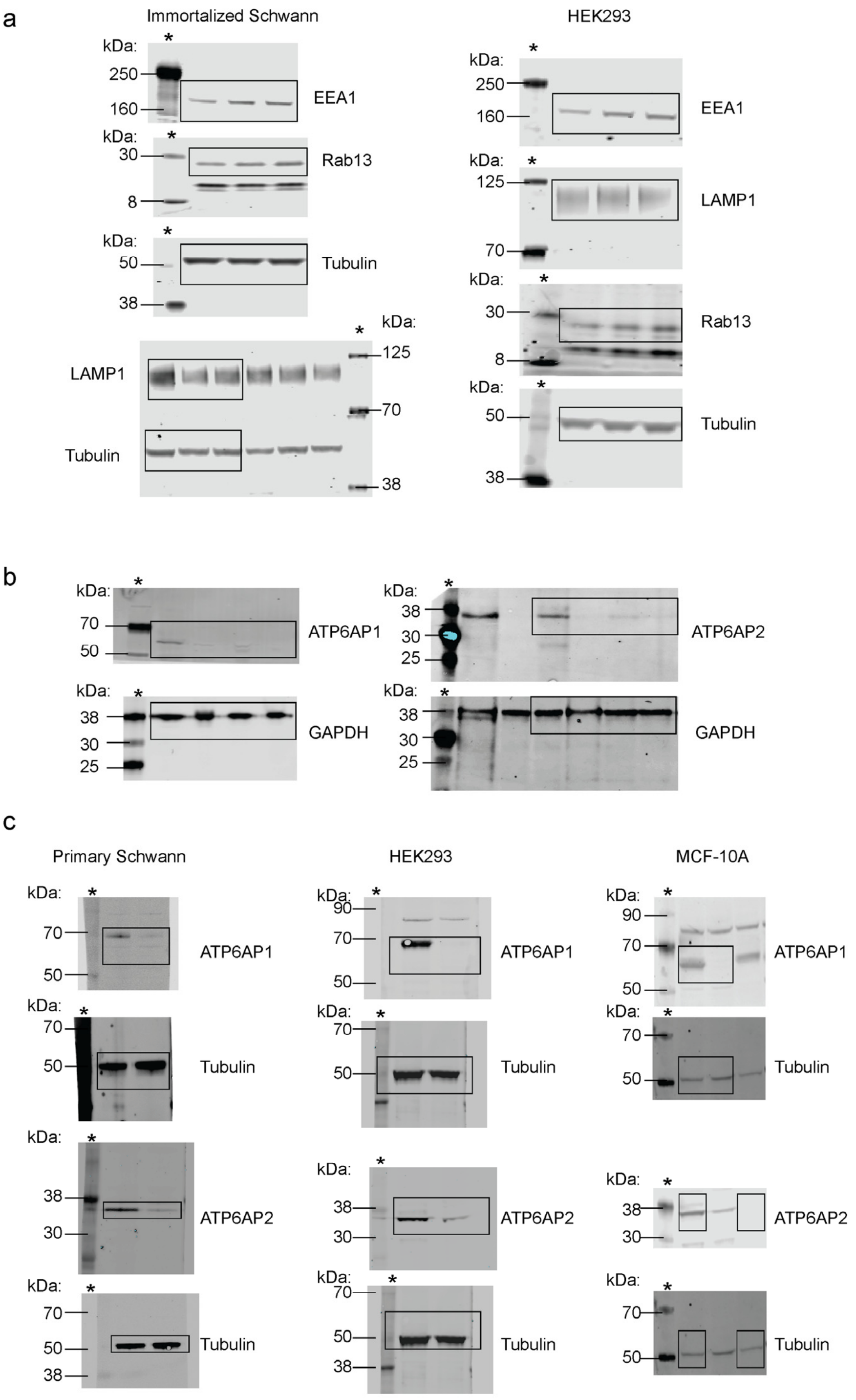

Supplementary Figure 6: Unprocessed images of western blots.
Unprocessed images of scanned immunoblots shown in **(a)** Supplementary Figure 4d, **(b)** Supplementary Figure 3b and **(c)** Supplementary Figure 4a of the manuscript are provided. Molecular weight ladders are indicated by an asterisk (*).

Supplementary Figure 7

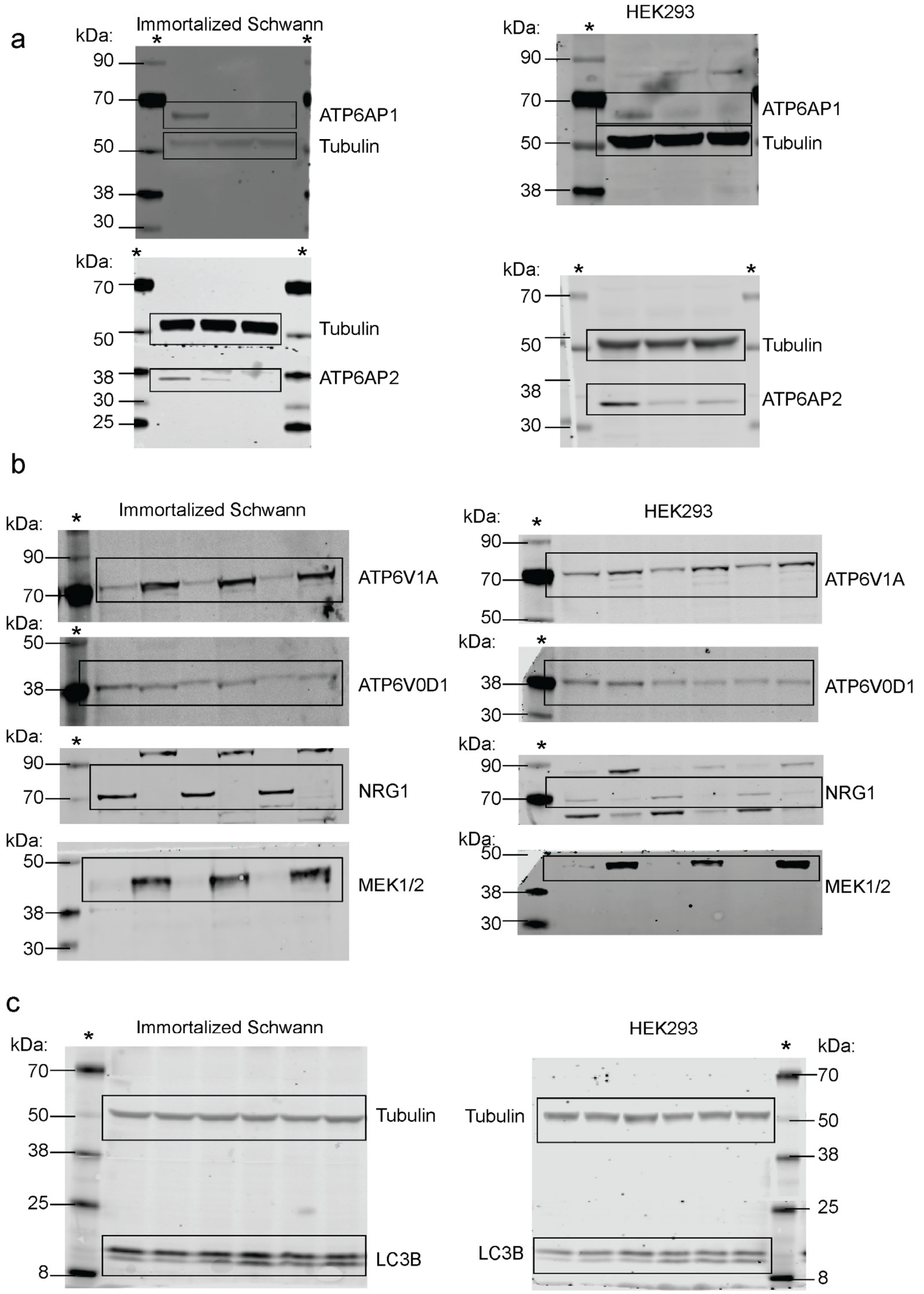

Supplementary Figure 7: Unprocessed images of western blots.
Unprocessed images of scanned immunoblots shown in **(a)** Supplementary Figure 4c, **(b)** Supplementary Figure 5a and **(c)** Supplementary Figure 5b of the manuscript are provided. Molecular weight ladders are indicated by an asterisk (*).

Supplementary Figure 8

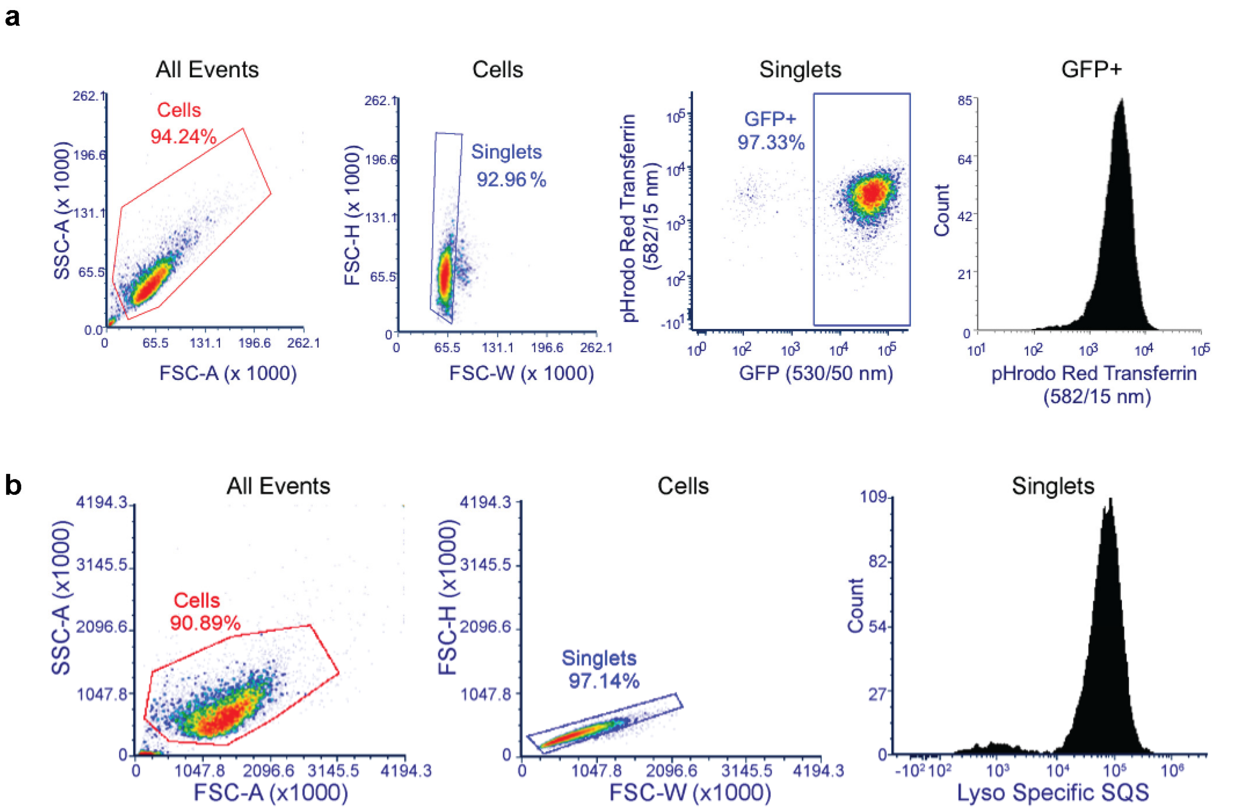

Supplementary Figure 8: Depiction of gating strategies employed for flow cytometry assays. Sequential plots illustrating the gating strategies used in **(a)** Supplementary Figure 4h-4i and **(b)** Figure 5c and Supplementary Figure 4g of the manuscript are provided.

Supplementary Table 1: Clinicopathologic and sequencing information of the granular cell tumors included in this study.

Case ID	Sex	Anatomic location	Mitosis >2	Spindling	Increased nuclear/cytoplasmic ratio	Vesicular nuclei and prominent nucleoli	Nuclear pleomorphism	Necrosis	Fanburg-Smith histologic classification	DNA sequencing platform	RNA sequencing	*ATP6AP1* mutation	*ATP6AP2* mutation	Mutation type	Validation by Sanger sequencing and/ or repeat targeted capture
GCT1	M	Soft tissue	No	Yes	No	Yes	No	No	Atypical	WES	Yes	WT	p.Q188*	Truncating SNV	*ATP6AP2* (p.Q188*)
GCT2	F	Gastrointestinal tract	Yes	Yes	Yes	Yes	Yes	Yes	Malignant	WES	Yes	p.L345Qfs*32	WT	Frame-shift indel	*ATP6AP1* (p.L345Qfs*32)
GCT3	F	Skin	No	No	No	No	No	No	Benign	WES	No	p.Q293*	WT	Truncating SNV	*ATP6AP1* (p.Q293*)
GCT4	M	Soft tissue	No	No	No	No	No	No	Benign	WES	Yes	WT	p.I313Dfs*22	Frame-shift indel	*ATP6AP2* (p.I313Dfs*22)
GCT5	F	Soft tissue	No	No	No	No	No	No	Benign	WES	Yes	p.T388Sfs*7	WT	Frame-shift indel	*ATP6AP1* (p.T388Sfs*7)
GCT6	F	Bladder	Yes	Yes	Yes	Yes	Yes	Yes	Malignant	WES	No	p.W138*	WT	Truncating SNV	*ATP6AP1* (p.W138*)
GCT8	F	Soft tissue	No	No	No	No	No	No	Benign	WES	No	WT	WT	N/A	N/A
GCT9	F	Gastrointestinal tract	No	No	No	No	No	No	Benign	WES	No	WT	p.P90Qfs*48	Frame-shift indel	*ATP6AP2* (p.P90Qfs*48)
GCT10	F	Gastrointestinal tract	No	Yes	No	No	No	No	Atypical	WES	Yes	p.L377*	WT	Frame-shift indel	*ATP6AP1* (p.L377*)
GCT11	F	Skin	No	No	No	No	No	No	Benign	WES	Yes	WT	WT	N/A	N/A
GCT12	M	Skin	No	No	No	Yes	No	No	Atypical	WES	Yes	WT	WT	N/A	N/A
GCT13	M	Soft tissue	No	No	No	No	No	No	Benign	WES	Yes	p.L430Cfs*17	WT	Frame-shift indel	*ATP6AP1* (p.L430Cfs*17)
GCT14	F	Soft tissue	No	No	No	No	No	No	Benign	WES	No	WT	WT	N/A	N/A
GCT15	F	Soft tissue	No	Yes	No	Yes	No	No	Atypical	WES	Yes	p.Q403Rfs*5	WT	Frame-shift indel	*ATP6AP1* (p.Q403Rfs*5)
GCT16	F	Soft tissue	No	No	No	No	No	No	Benign	WES	Yes	p.W393*	WT	Truncating SNV	*ATP6AP1* (p.W393*)
GCT18	F	Skin	No	No	No	No	No	No	Benign	Targeted capture	No	WT	p.Q160*	Truncating SNV	*ATP6AP2* (p.Q160*)
GCT20	F	Soft tissue	No	No	No	No	No	No	Benign	WES	No	WT	WT	N/A	N/A
GCT21	F	Skin	No	No	No	No	No	No	Benign	Targeted capture	No	p.Q401Rfs*7	WT	Frame-shift indel	*ATP6AP1* (p.Q401Rfs*7)
GCT22	F	Soft tissue	No	No	No	No	No	No	Benign	Targeted capture	No	p.Y147*	WT	Truncating SNV	*ATP6AP1* (p.Y147*)
GCT23	F	Skin	No	No	No	No	No	No	Benign	Targeted capture	Yes	WT	WT	N/A	N/A
GCT25	F	Breast	No	No	No	Yes	Yes	No	Atypical	Targeted capture	No	p.S368del	WT	In-frame indel	*ATP6AP1* (p.S368del)
GCT28	F	Gastrointestinal tract	No	No	No	No	No	No	Benign	Targeted capture	No	p.H67Sfs*11	WT	Frame-shift indel	*ATP6AP1* (p.H67Sfs*11)
GCT32	F	Skin	No	No	No	No	Yes	No	Atypical	Targeted capture	No	p.L82Wfs*16	WT	Frame-shift indel	*ATP6AP1* (p.L82Wfs*16)
GCT35	F	Lung	No	No	No	No	No	No	Benign	Targeted capture	No	WT	WT	N/A	N/A
GCT36	F	Soft tissue	No	No	No	No	No	No	Benign	Targeted capture	No	p.W50*	WT	Truncating SNV	*ATP6AP1* (p.W50*)
GCT37	M	Skin	Yes	Yes	No	No	No	No	Atypical	Targeted capture	No	WT	WT	N/A	N/A
GCT38	F	Breast	No	Yes	No	No	No	No	Atypical	Targeted capture	No	WT	p.R31*	Truncating SNV	*ATP6AP2* (p.R31*)
GCT39	F	Soft tissue	No	No	No	No	Yes	No	Atypical	Targeted capture	No	p.F274Sfs*12	WT	Frame-shift indel	*ATP6AP1* (p.F274Sfs*12)
GCT40	F	Gastrointestinal tract	No	Yes	No	No	No	No	Atypical	Targeted capture	No	WT	WT	N/A	N/A
GCT41	F	Tongue	No	Yes	No	No	No	No	Atypical	Targeted capture	No	p.X324_splice	WT	Splice site SNV	*ATP6AP1* (p.X324_splice)
GCT42	M	Soft tissue	No	No	No	No	No	No	Benign	Targeted capture	No	p.X76_splice	WT	Splice site SNV	*ATP6AP1* (p.X76_splice)
GCT43	F	Gastrointestinal tract	No	Yes	No	No	No	No	Atypical	Targeted capture	No	p.X82_splice	WT	Splice site SNV	*ATP6AP1* (p.X82_splice)
GCT44	F	Soft tissue	No	No	No	No	No	No	Benign	Targeted capture	No	p.F274del	WT	In-frame indel	*ATP6AP1* (p.F274del)
GCT45	F	Gastrointestinal tract	No	Yes	No	No	No	No	Atypical	Targeted capture	No	p.L331Pfs*46	WT	Frame-shift indel	*ATP6AP1* (p.L331Pfs*46)
GCT46	F	Gastrointestinal tract	No	Yes	No	Yes	No	No	Atypical	Targeted capture	No	WT	WT	N/A	N/A
GCT47	F	Breast	No	No	No	Yes	Yes	No	Atypical	Targeted capture	No	p.W21Vfs*29	WT	Frame-shift indel	*ATP6AP1* (p.W21Vfs*29)
GCT48	F	Breast	No	No	Yes	Yes	Yes	No	Malignant	Targeted capture	No	p.Q44Rfs*39	WT	Frame-shift indel	*ATP6AP1* (p.Q44Rfs*39)
GCT49	M	Gastrointestinal tract	No	Yes	No	Yes	Yes	No	Malignant	Targeted capture	No	p.S382Gfs*72	WT	Frame-shift indel	*ATP6AP1* (p.S382Gfs*72)
GCT52	F	Tongue	No	No	No	No	No	No	Benign	Targeted capture	No	WT	WT	N/A	N/A
GCT53	F	Breast	No	No	No	No	No	No	Benign	Targeted capture	No	p.P46Afs*5	WT	Frame-shift indel	*ATP6AP1* (p.P46Afs*5)
GCT54	F	Tongue	No	No	No	No	No	No	Benign	Targeted capture	No	WT	p.D107Vfs*29	Frame-shift indel	*ATP6AP2* (p.D107Vfs*29)
GCT55	F	Skin	No	No	No	No	No	No	Benign	Targeted capture	No	p.G425Rfs*33	WT	Frame-shift indel	*ATP6AP1* (p.G425Rfs*33)
GCT57	F	Skin	No	No	No	No	No	No	Benign	Targeted capture	No	WT	p.N258Sfs*47	Frame-shift indel	*ATP6AP2* (p.N258Sfs*47)
GCT58	F	Skin	No	No	No	No	No	No	Benign	Targeted capture	No	p.F422Sfs*10	WT	Frame-shift indel	*ATP6AP1* (p.F422Sfs*10)
GCT59	M	Skin	No	No	No	No	No	No	Benign	Targeted capture	No	WT	p.L68Cfs*11	Frame-shift indel	*ATP6AP2* (p.L68Cfs*11)
GCT60	F	Gastrointestinal tract	No	Yes	No	No	No	No	Atypical	Targeted capture	No	p.Q403Pfs*3	WT	Frame-shift indel	*ATP6AP1* (p.Q403Pfs*3)
GCT61	F	Skin	No	No	No	No	No	No	Benign	Targeted capture	No	p.L72Cfs*7	WT	Frame-shift indel	*ATP6AP1* (p.L72Cfs*7)
GCT62	F	Skin	No	Yes	No	No	Yes	No	Atypical	Targeted capture	No	WT	WT	N/A	N/A
GCT63	M	Skin	No	No	No	No	No	No	Benign	Targeted capture	No	p.F356Sfs*22	WT	Frame-shift indel	*ATP6AP1* (p.F356Sfs*22)
GCT64	F	Skin	No	No	No	No	No	No	Benign	Targeted capture	No	p.X200_splice	WT	Splice site SNV	*ATP6AP1* (p.X200_splice)
GCT65	F	Skin	No	No	No	No	No	No	Benign	Targeted capture	No	p.W427*	WT	Truncating SNV	*ATP6AP1* (p.W427*)
GCT66	M	Skin	No	No	No	No	No	No	Benign	Targeted capture	No	p.N296Ifs*4	WT	Frame-shift indel	*ATP6AP1* (p.N296Ifs*4)
GCT67	F	Skin	No	No	No	Yes	No	No	Atypical	Targeted capture	No	p.Y414_S416del	WT	In-frame indel	*ATP6AP1* (p.Y414_S416del)
GCT68	F	Skin	No	No	No	No	No	No	Benign	Targeted capture	No	WT	WT	N/A	N/A
GCT69	F	Skin	No	No	No	Yes	No	No	Atypical	Targeted capture	No	WT	WT	N/A	N/A
GCT70	F	Skin	No	No	No	No	No	No	Benign	Targeted capture	No	p.S335_E343del	WT	In-frame indel	*ATP6AP1* (p.S335_E343del)
GCT71	F	Skin	No	No	No	No	No	No	Benign	Targeted capture	No	p.W427Hfs*22	WT	Frame-shift indel	*ATP6AP1* (p.W427Hfs*22)
GCT72	F	Soft tissue	No	Yes	No	No	Yes	No	Atypical	Targeted capture	No	p.W57*	WT	Truncating SNV	*ATP6AP1* (p.W57*)
GCT73	F	Skin	No	No	No	Yes	No	No	Atypical	Targeted capture	No	p.X308_splice	WT	Splice site SNV	*ATP6AP1* (p.X308_splice)
GCT74	F	Skin	Yes	Yes	No	No	No	No	Atypical	Targeted capture	No	WT	WT	N/A	N/A
GCT75	M	Skin	No	Yes	No	No	No	No	Atypical	Targeted capture	No	p.S129Pfs*16	WT	Frame-shift indel	*ATP6AP1* (p.S129Pfs*16)
GCT78	F	Skin	No	No	No	No	No	No	Benign	Targeted capture	No	p.A354Lfs*20	WT	Frame-shift indel	*ATP6AP1* (p.A354Lfs*20)
GCT79	F	Skin	No	No	No	No	No	No	Benign	Targeted capture	No	p.N111Tfs*10	WT	Frame-shift indel	*ATP6AP1* (p.N111Tfs*10)
GCT80	F	Skin	No	No	No	No	No	No	Benign	Targeted capture	No	WT	WT	N/A	N/A
GCT81	F	Breast	No	Yes	No	Yes	No	No	Atypical	Targeted capture	No	p.R87Sfs*6	WT	Frame-shift indel	*ATP6AP1* (p.R87Sfs*6)
GCT82	F	Skin	No	No	No	Yes	No	No	Atypical	Targeted capture	No	p.S70Afs*9	WT	Frame-shift indel	*ATP6AP1* (p.S70Afs*9)
GCT83	F	Skin	No	No	No	No	No	No	Benign	Targeted capture	No	p.Y414Sfs*25	WT	Frame-shift indel	*ATP6AP1* (p.Y414Sfs*25)
GCT84	F	Skin	No	Yes	Yes	No	Yes	No	Malignant	Targeted capture	No	WT	WT	N/A	N/A
GCT85	F	Gastrointestinal tract	No	Yes	Yes	No	No	No	Atypical	Targeted capture	No	WT	WT	N/A	N/A

GCT86	F	Gastrointestinal tract	No	Yes	Yes	No	No	No	Atypical	Targeted capture	No	p.R315*	WT	Truncating SNV	*ATP6AP1* (p.R315*)
GCT87	F	Skin	No	No	No	No	No	No	Benign	Targeted capture	No	WT	WT	N/A	N/A
GCT88	F	Skin	No	No	No	Yes	Yes	No	Atypical	Targeted capture	No	p.V48Tfs*26	WT	Frame-shift indel	*ATP6AP1* (p.V48Tfs*26)
GCT89	F	Skin	Yes	Yes	No	Yes	Yes	No	Malignant	Targeted capture	No	p.X200_splice	WT	Splice site SNV	*ATP6AP1* (p.X200_splice)
GCT90	F	Skin	No	No	No	No	No	No	Benign	Targeted capture	No	WT	WT	N/A	N/A
GCT91	F	Skin	No	No	No	Yes	No	No	Atypical	Targeted capture	No	p.S382Vfs*14	WT	Frame-shift indel	*ATP6AP1* (p.S382Vfs*14)
GCT92	F	Gastrointestinal tract	No	Yes	No	No	No	No	Atypical	WES	No	p.W302*	WT	Truncating SNV	*ATP6AP1* (p.W302*)
GCT93	F	Parotid gland	No	No	No	No	No	No	Benign	Targeted capture	No	WT	WT	N/A	N/A
GCT95	M	Tongue	No	No	No	No	No	No	Benign	Targeted capture	No	WT	WT	N/A	N/A
GCT96	F	Skin	No	No	No	No	No	No	Benign	Targeted capture	No	WT	WT	N/A	N/A
GCT97	M	Skin	No	No	Yes	No	No	No	Atypical	Targeted capture	No	WT	p.X95_splice	Splice site SNV	*ATP6AP2* (p.X95_splice)
GCT98	F	Skin	No	No	No	No	No	No	Benign	Targeted capture	No	p.M395del	WT	In-frame indel	*ATP6AP1* (p.M395del)
GCT99	F	Tongue	No	Yes	No	No	No	No	Atypical	Targeted capture	No	p.A135Pfs*10	WT	Frame-shift indel	*ATP6AP1* (p.A135Pfs*10)

F, female; M, male; N/A, not applicable; WES, whole-exome sequencing; WT, wild-type

Supplementary Table 2: List of fusion genes and/or readthroughs identified by RNA-sequencing analysis of granular cell tumors.

Sample	Gene 5'	Gene 3'	Mapping 5'	Mapping 3'	Total reads	Spanning reads	In-frame	Read-through	Driver Probability (Oncofuse)
GCT15T	DHTKD1	SEC61A2	chr10:12162267	chr10:12175239	4	2	No	No	0.0168
GCT23T	ZNF841	ZNF432	chr19:52592189	chr19:52550314	17	5	Yes	No	0.9737
GCT2T	FAM214A	ARPP19	chr15:52876940	chr15:52849419	4	2	No	Yes	0.0416
GCT1T	RRP7B	KCTD19	chr22:42961107	chr16:67355624	9	4	No	No	0.0471
GCT23T	RRP7B	KCTD19	chr22:42961107	chr16:67355624	9	4	No	No	0.0471
GCT23T	7SK/IL1RAP	KCTD19	chr3:190360198	chr16:67355618	8	5	No	Yes	0.1134

Supplementary Table 3: Sequencing statistics of the granular cell tumors subjected to whole-exome sequencing.

Case	Tissue Type (Sample ID)	Total Reads	Mean Target Coverage	Target Bases 2X	Target Bases 50X	Target Bases 100X
GCT1	Tumor (GCT1T)	85,063,839	99.21	99.63%	83.01%	43.44%
	Normal (GCT1N)	77,362,194	92.60	99.65%	79.95%	38.34%
GCT2	Tumor (GCT2T)	128,101,984	150.61	99.71%	90.05%	65.69%
	Normal (GCT2N)	162,779,641	193.52	99.81%	92.35%	75.98%
GCT3	Tumor (GCT3T)	79,728,767	96.03	99.78%	81.29%	40.22%
	Normal (GCT3N)	65,949,245	73.98	99.71%	71.35%	22.44%
GCT4	Tumor (GCT4T)	109,135,641	114.36	99.55%	87.29%	51.90%
	Normal (GCT4N)	86,170,100	93.40	99.67%	81.55%	38.69%
GCT5	Tumor (GCT5T)	122,861,678	157.33	99.78%	91.56%	69.28%
	Normal (GCT5N4)	91,559,376	78.83	99.58%	72.69%	27.14%
GCT6	Tumor (GCT6T)	109,400,409	125.72	99.78%	78.74%	49.44%
	Normal (GCT6N)	75,155,499	86.84	99.74%	66.29%	31.43%
GCT8	Tumor (GCT8T)	123,061,507	151.53	99.79%	90.63%	66.16%
	Normal (GCT8N)	78,553,308	94.83	99.74%	76.46%	36.71%
GCT9	Tumor (GCT9T)	99,405,995	117.89	99.77%	85.17%	51.96%
	Normal (GCT9N)	96,023,264	117.44	99.79%	79.47%	46.40%
GCT10	Tumor (GCT10T)	129,047,709	111.21	99.78%	76.88%	43.78%
	Normal (GCT10N)	77,971,965	92.88	99.77%	69.11%	34.64%
GCT11	Tumor (GCT11T3)	105,270,395	102.91	99.68%	75.61%	40.05%
	Normal (GCT11N3)	94,293,572	72.85	99.48%	70.98%	22.06%
GCT12	Tumor (GCT12T3)	117,359,514	106.59	99.63%	81.15%	45.35%
	Normal (GCT12N3)	109,533,044	115.10	99.66%	81.62%	48.59%
GCT13	Tumor (GCT13T2)	103,400,606	98.60	99.47%	83.17%	44.71%
	Normal (GCT13N2)	93,364,190	86.17	99.43%	78.04%	34.38%
GCT14	Tumor (GCT14T2)	102,990,531	75.37	99.40%	72.51%	24.46%
	Normal (GCT14N2)	107,306,446	96.19	99.56%	68.84%	36.82%
GCT15	Tumor (GCT15T2)	157,583,873	118.84	99.67%	84.52%	51.95%
	Normal (GCT15N2)	102,265,966	86.67	99.61%	60.47%	31.52%
GCT16	Tumor (GCT16T2)	113,352,436	115.10	99.71%	77.41%	45.35%
	Normal (GCT16N2)	95,610,219	82.69	99.56%	72.11%	30.48%
GCT20	Tumor (GCT20T2)	108,812,267	119.65	99.57%	87.08%	56.94%
	Normal (GCT20N2)	75,437,421	66.22	99.33%	65.43%	16.04%
GCT92	Tumor (GCT92T)	79,293,291	91.63	99.69%	78.34%	36.57%
	Normal (GCT92N)	94,268,623	118.34	99.64%	86.88%	55.27%

Supplementary Table 4: List of non-synonymous somatic mutations identified in granular cell tumors by whole-exome sequencing.

Sample	Gene	Amino Acid Change	Chromosome	Position	Reference Allele	Alternate Allele	Effect	Tumor MAF	Normal MAF	Tumor Depth	Normal Depth	Cancer Cell Fraction	Clonal Probability	95% Confidence Interval High	95% Confidence Interval Low	Clonality	Exac Score
GCT10T	ATP6AP1	p.L377*	23	153663775	GC	G	Frame_Shift_Del	0.333333333	0	270	232	1	0.97403	1	0.936201946	Clonal	.
GCT10T	TPRXL	p.S212P	3	14106310	T	C	Missense_Mutation	0.090909091	0	44	50	0.54	0.283	0.96047682	0.210705647	Subclonal	.
GCT10T	NAMPTL	p.V77F	10	36812934	C	A	Missense_Mutation	0.114285714	0	35	44	0.68	0.4316	0.978386113	0.253973488	Subclonal	.
GCT10T	HSPB8	p.N184S	12	119631623	A	G	Missense_Mutation	0.069767442	0	43	35	0.42	0.16482	0.928331337	0.144339435	Subclonal	.
GCT10T	ATP5EP2	p.V22A	13	28519461	T	C	Missense_Mutation	0.072072072	0	111	90	0.43	0.02192	0.801573918	0.219054293	Subclonal	.
GCT10T	CCDC40	p.T931M	17	78063643	C	T	Missense_Mutation	0.246575342	0	73	55	1	0.85751	1	0.707663136	Clonal	1.89E-05
GCT11T3	IK	p.E94Afs*21	5	140032592	TGA	T	Frame_Shift_Del	0.157894737	0	19	12	1	0.75421	0.989909629	0.303173376	Clonal	.
GCT11T3	ZC3H3	p.S881del	8	144522386	TGAG	T	In_Frame_Del	0.173913043	0	23	8	1	0.79972	1	0.375851262	Clonal	.
GCT11T3	FAM155A	p.Q86del	13	108518686	TCTG	T	In_Frame_Del	0.086956522	0	23	9	0.76	0.5782	0.981211758	0.177179513	Clonal	.
GCT11T3	UBE2J2	p.I130V	1	1192446	T	C	Missense_Mutation	0.104477612	0	67	33	0.91	0.6792	0.988751121	0.399028588	Clonal	.
GCT11T3	PLXND1	p.Y978C	3	129291689	T	C	Missense_Mutation	0.147058824	0	102	57	1	0.84705	1	0.632127852	Clonal	.
GCT11T3	SMC4	p.R932K	3	160146730	G	A	Missense_Mutation	0.070175439	0	57	52	0.61	0.45409	0.972117205	0.230663687	Subclonal	.
GCT11T3	FAM71B	p.R141C	5	156592759	G	A	Missense_Mutation	0.134020619	0.016949153	97	59	1	0.8147	1	0.580422007	Clonal	5.65E-05
GCT11T3	DENND1A	p.R448H	9	126212985	C	T	Missense_Mutation	0.204545455	0	44	45	1	0.90376	1	0.588003847	Clonal	9.42E-06
GCT11T3	OR5AS1	p.L29F	11	55797981	G	C	Missense_Mutation	0.192307692	0	52	58	1	0.89942	1	0.602363549	Clonal	9.43E-06
GCT11T3	FAT3	p.V547F	11	92086917	G	T	Missense_Mutation	0.150943396	0	53	62	1	0.81593	1	0.509269539	Clonal	.
GCT11T3	AHNAK2	p.T1283M	14	105417940	G	A	Missense_Mutation	0.266666667	0	30	19	1	0.90179	1	0.592758526	Clonal	5.50E-05
GCT11T3	ITGB1BP2	p.T232N	23	70524092	C	A	Missense_Mutation	0.131578947	0	76	49	1	0.87407	1	0.583555682	Clonal	.
GCT11T3	FRMPD3	p.R1268W	23	106844972	C	T	Missense_Mutation	0.203488372	0	172	63	1	0.91957	1	0.860156443	Clonal	.
GCT11T3	NAALAD2	p.X678_splice	11	89916178	T	A	Splice_Site	0.098360656	0.016666667	61	60	0.86	0.64391	0.98682284	0.358373065	Clonal	.
GCT12T3	RHBG	p.R425Dfs*35	1	156354347	TC	T	Frame_Shift_Del	0.096774194	0	31	33	0.67	0.44692	0.978124025	0.213207815	Subclonal	.
GCT12T3	KCTD16	p.A384Lfs*20	5	143853530	CA	C	Frame_Shift_Del	0.102564103	0	39	39	0.71	0.46824	0.980069696	0.260006725	Subclonal	9.70E-04
GCT12T3	GRM1	p.P842Lfs*76	6	146720698	GC	G	Frame_Shift_Del	0.118421053	0.010526316	76	95	0.82	0.55849	0.986197096	0.413339347	Clonal	.
GCT12T3	SRRM3	p.G326Afs*323	7	75896717	CG	C	Frame_Shift_Del	0.111111111	0	18	21	0.88	0.5481	0.983721653	0.191652668	Clonal	.
GCT12T3	GID4	p.D81Tfs*61	17	17943014	CG	C	Frame_Shift_Del	0.1	0	20	22	0.79	0.51601	0.982074013	0.181953057	Clonal	.
GCT12T3	LMAN1	p.E305Rfs*20	18	57013193	C	CT	Frame_Shift_Ins	0.08	0	25	23	0.55	0.38416	0.971689826	0.14653981	Subclonal	1.13E-04
GCT12T3	TTC13	p.C11del	1	231114542	AAGC	A	In_Frame_Del	0.117647059	0	17	23	0.81	0.52527	0.982564098	0.185156693	Clonal	4.63E-04
GCT12T3	PVRIG	p.R95H	7	99817902	G	A	Missense_Mutation	0.105263158	0	38	32	0.83	0.54559	0.9850921	0.288350817	Clonal	9.75E-06
GCT12T3	MAP3K9	p.A39E	14	71275773	G	T	Missense_Mutation	0.115384615	0	26	41	0.8	0.52311	0.98334483	0.238880762	Clonal	.
GCT12T3	SEZ6L	p.T405M	22	26695001	C	T	Missense_Mutation	0.104166667	0	48	59	0.72	0.47209	0.980503969	0.293506996	Subclonal	5.65E-05
GCT12T3	ZRANB2	p.R33*	1	71544351	G	A	Nonsense_Mutation	0.166666667	0	18	16	1	0.64853	0.988693968	0.286711032	Clonal	.
GCT13T2	ASXL2	p.A636Pfs*135	2	25967216	CT	C	Frame_Shift_Del	0.102362205	0	127	91	1	0.77873	1	0.539572495	Clonal	.
GCT13T2	ATP6AP1	p.L430Cfs*17	23	153664111	GC	G	Frame_Shift_Del	0.4	0.012987013	80	77	1	0.54448	1	0.867169355	Clonal	.
GCT13T2	RMDN3	p.T386Nfs*7	15	41029893	G	GT	Frame_Shift_Ins	0.083333333	0	24	28	0.83	0.61868	0.982843023	0.185456693	Clonal	.
GCT13T2	NANOS1	p.D112del	10	120789634	GGAC	G	In_Frame_Del	0.142857143	0	14	10	1	0.73442	0.987914254	0.22757665	Clonal	.
GCT13T2	BPTF	p.E148del	17	65822266	CGAG	C	In_Frame_Del	0.081081081	0	37	25	0.81	0.61462	0.983588241	0.238524679	Clonal	.
GCT13T2	NAP1L2	p.E221del	23	72433663	GTCC	G	In_Frame_Del	0.133333333	0	45	41	1	0.80663	1	0.446692097	Clonal	.
GCT13T2	DNAH14	p.P1095L	1	225270398	C	T	Missense_Mutation	0.1	0	30	31	1	0.68297	0.986999958	0.26598345	Clonal	5.25E-05
GCT13T2	SEMA3G	p.P97L	3	52476624	G	A	Missense_Mutation	0.073170732	0	41	17	0.73	0.57462	0.981063602	0.224093738	Clonal	9.42E-06
GCT13T2	GIMAP1	p.V13F	7	150416172	G	T	Missense_Mutation	0.181818182	0	55	39	1	0.9156	1	0.612230092	Clonal	.
GCT13T2	ZNF317	p.G424R	19	9271591	G	A	Missense_Mutation	0.118421053	0.02173913	76	46	1	0.79874	1	0.509267992	Clonal	.
GCT13T2	CYP2A6	p.F384Y	19	41351209	A	T	Missense_Mutation	0.166666667	0.03125	30	32	1	0.84383	1	0.437275847	Clonal	.
GCT13T2	DDX53	p.A43T	23	23018301	G	A	Missense_Mutation	0.076923077	0	52	42	0.77	0.59542	0.982879527	0.272083844	Clonal	.
GCT13T2	SLC9A2	p.R480*	2	103310885	C	T	Nonsense_Mutation	0.27027027	0	37	57	1	0.86763	1	0.656863697	Clonal	.
GCT14T2	KCNN3	p.P112Lfs*64	1	154842105	AG	A	Frame_Shift_Del	0.125	0	16	70	1	0.65715	0.9855833	0.205060083	Clonal	.
GCT14T2	IFNA2	p.I170Tfs*2	9	21384819	GA	G	Frame_Shift_Del	0.161290323	0	31	10	1	0.78222	1	0.40939742	Clonal	.
GCT14T2	LMAN1	p.E305Rfs*22	18	57013193	CT	C	Frame_Shift_Del	0.125	0	16	22	1	0.65715	0.9855833	0.205060083	Clonal	.
GCT14T2	COBLL1	p.L907Ffs*25	2	165551295	C	CA	Frame_Shift_Ins	0.185185185	0	27	15	1	0.81738	1	0.429342127	Clonal	.
GCT14T2	TTK	p.R854Kfs*11	6	80751896	G	GA	Frame_Shift_Ins	0.178571429	0	28	8	1	0.80873	1	0.42425105	Clonal	.
GCT14T2	EP400	p.H31Pfs*34	12	132445252	G	GC	Frame_Shift_Ins	0.125	0	16	16	1	0.65715	0.9855833	0.205060083	Clonal	.
GCT14T2	HOXB3	p.Q34Pfs*68	17	46629736	T	TG	Frame_Shift_Ins	0.129032258	0	31	123	1	0.70191	0.989147329	0.328786045	Clonal	.
GCT14T2	ALS2CL	p.L89del	3	46729621	CGCA	C	In_Frame_Del	0.095238095	0	21	38	0.79	0.58392	0.981940849	0.181114562	Clonal	.
GCT14T2	GOLIM4	p.Q453del	3	167747641	CCTG	C	In_Frame_Del	0.108108108	0	37	55	0.89	0.64163	0.986514294	0.299945221	Clonal	0.002759
GCT14T2	FAM8A1	p.R115del	6	17600982	CCGG	C	In_Frame_Del	0.121212121	0.006849315	33	146	1	0.68188	0.988356998	0.318918512	Clonal	.
GCT14T2	NFE2L3	p.D229del	7	26217668	AATG	A	In_Frame_Del	0.105263158	0	19	13	0.87	0.61316	0.98353568	0.190359329	Clonal	2.83E-05
GCT14T2	DAB2IP	p.A89del	9	124461707	GCGC	G	In_Frame_Del	0.176470588	0.01754386	17	57	1	0.76041	1	0.309672904	Clonal	.
GCT14T2	IRF2BPL	p.A164del	14	77493647	AGCG	A	In_Frame_Del	0.25	0	8	13	1	0.75984	0.989554337	0.250182753	Clonal	.
GCT14T2	EIF5	p.P185del	14	103804756	ACAC	A	In_Frame_Del	0.066666667	0	30	53	0.55	0.45213	0.971626987	0.145556848	Subclonal	.
GCT14T2	ALG1	p.L20del	16	5121897	CGCT	C	In_Frame_Del	0.086956522	0	23	41	0.72	0.55474	0.98012725	0.172361845	Clonal	9.48E-06
GCT14T2	DNM3	p.I667L	1	172348263	A	C	Missense_Mutation	0.083333333	0	60	19	0.69	0.51312	0.978240295	0.282164783	Clonal	.
GCT14T2	NEB	p.R2691C	2	152499753	G	A	Missense_Mutation	0.088235294	0	34	30	0.73	0.55579	0.98098742	0.224646815	Clonal	4.73E-05
GCT14T2	SNCAIP	p.G480R	5	121780273	G	A	Missense_Mutation	0.176470588	0	17	20	1	0.76041	1	0.309672904	Clonal	.
GCT14T2	C7orf55-LUC7L2	p.S2L	7	139045012	C	T	Missense_Mutation	0.096774194	0	31	56	0.8	0.59264	0.983341195	0.237808951	Clonal	.
GCT14T2	RIMBP2	p.R119W	12	130935838	G	A	Missense_Mutation	0.083333333	0.011494253	36	87	0.69	0.5307	0.979174662	0.216361068	Clonal	9.42E-06
GCT14T2	NAGLU	p.G506R	17	40695540	G	A	Missense_Mutation	0.176470588	0	17	36	1	0.76041	1	0.309672904	Clonal	.
GCT14T2	P2RX6	p.V339I	22	21380330	G	A	Missense_Mutation	0.09375	0	32	109	0.77	0.58045	0.982603077	0.2333184	Clonal	.
GCT15T2	PABPC3	p.F335Lfs*19	13	25671332	CA	C	Frame_Shift_Del	0.117647059	0	17	14	1	0.69156	0.98699784	0.217444822	Clonal	.
GCT15T2	NUTM1	p.R345Afs*24	15	34646682	GC	G	Frame_Shift_Del	0.090909091	0	22	18	0.95	0.63521	0.9846676	0.197305375	Clonal	.
GCT15T2	ATP6AP1	p.Q403Rfs*5	23	153664029	TC	T	Frame_Shift_Del	0.206751055	0.004716981	237	212	1	0.91221	1	0.894948129	Clonal	.
GCT15T2	GTF3C3	p.T361Nfs*11	2	197649613	G	GT	Frame_Shift_Ins	0.090909091	0	33	16	0.95	0.65037	0.986397535	0.260111102	Clonal	.
GCT15T2	SMAP1	p.E173Gfs*19	6	71508369	G	GA	Frame_Shift_Ins	0.097560976	0	41	10	1	0.68612	0.988573474	0.320408847	Clonal	.
GCT15T2	MYO6	p.Q918Tfs*24	6	76599857	G	GA	Frame_Shift_Ins	0.089285714	0	56	22	0.94	0.66634	0.988148869	0.345105891	Clonal	.
GCT15T2	FLCN	p.H429Pfs*27	17	17119708	T	TG	Frame_Shift_Ins	0.102564103	0	39	40	1	0.70188	0.989173688	0.328045674	Clonal	.
GCT15T2	RXRB	p.A52del	6	33168096	ACCG	A	In_Frame_Del	0.125	0	24	27	1	0.73468	0.989525995	0.296720575	Clonal	.
GCT15T2	IRX5	p.A78del	16	54965326	GGCC	G	In_Frame_Del	0.096774194	0	31	31	1	0.66915	0.987197374	0.267844095	Clonal	1.49E-04
GCT15T2	SLC9A5	p.E708del	16	67300016	TGAG	T	In_Frame_Del	0.114285714	0	35	24	1	0.73359	1	0.343780965	Clonal	0.009918
GCT15T2	MED15	p.Q188del	22	20918816	ACAG	A	In_Frame_Del	0.151515152	0	33	21	1	0.82199	1	0.430977224	Clonal	.
GCT15T2	ZBED6	p.K121Q	1	203767011	A	C	Missense_Mutation	0.095652174	0	115	42	1	0.7441	1	0.50395304	Clonal	.
GCT15T2	GFPT1	p.R452C	2	69565158	G	A	Missense_Mutation	0.056179775	0	89	46	0.59	0.39519	0.966508135	0.247557263	Subclonal	9.42E-06

GCT15T2	RP11-766F14.2	p.T444K	4	100574475	G	T	Missense_Mutation	0.108333333	0	120	120	1	0.80093	1	0.570742128	Clonal	.
GCT15T2	FOPNL	p.R40C	16	15977973	G	A	Missense_Mutation	0.085106383	0	47	22	0.89	0.63867	0.986463449	0.298171414	Clonal	1.88E-05
GCT15T2	FITM2	p.G232R	20	42935360	C	T	Missense_Mutation	0.076923077	0	104	78	0.81	0.61015	0.985462262	0.387194956	Clonal	9.42E-06
GCT15T2	TSPEAR	p.T270M	21	45948448	G	A	Missense_Mutation	0.096774194	0	93	92	1	0.73383	1	0.470865756	Clonal	5.08E-04
GCT15T2	CD36		7	80293813	G	GGTAA	Nonsense_Mutation	0.387755102	0	49	16	1	0.05708	1	0.807171676	Subclonal	0.004567
GCT16T2	FRAS1	p.S1743Pfs*10	4	79359731	CT	C	Frame_Shift_Del	0.131147541	0	61	58	1	0.70989	1	0.459848457	Clonal	.
GCT16T2	KCNN3	p.Q41del	1	154842330	TTGC	T	In_Frame_Del	0.107142857	0	56	19	0.86	0.60187	0.986817671	0.359007459	Clonal	.
GCT16T2	YEATS2	p.G814del	3	183493743	CGGA	C	In_Frame_Del	0.24	0	25	23	1	0.86294	1	0.502498738	Clonal	.
GCT16T2	AARD	p.A88del	8	117950724	AGGC	A	In_Frame_Del	0.121212121	0	33	13	0.97	0.63552	0.987877639	0.313618027	Clonal	.
GCT16T2	FOXB2	p.A234del	9	79635238	TGGC	T	In_Frame_Del	0.095238095	0	21	13	0.76	0.53487	0.981306022	0.177979595	Clonal	.
GCT16T2	SFMBT2	p.V452_E457del	10	7247850	ACATCAACAAT	T	In_Frame_Del	0.06779661	0	59	61	0.54	0.32388	0.960812407	0.208605723	Subclonal	.
GCT16T2	ZIC2	p.A33del	13	100634393	CGCG	C	In_Frame_Del	0.114285714	0	35	21	0.91	0.61352	0.98691458	0.303773517	Clonal	.
GCT16T2	ERN1	p.L10del	17	62207359	TCAG	T	In_Frame_Del	0.166666667	0	12	11	1	0.67822	0.987604812	0.224498551	Clonal	8.61E-04
GCT16T2	ABCG5	p.P161L	2	44058927	G	A	Missense_Mutation	0.063492063	0	63	30	0.51	0.27462	0.95185581	0.196678008	Subclonal	.
GCT16T2	MUC2	p.T1758I	11	1093454	C	T	Missense_Mutation	0.068965517	0	87	52	0.55	0.263	0.950259987	0.251534186	Subclonal	8.70E-05
GCT16T2	FAT3	p.V611A	11	92087110	T	C	Missense_Mutation	0.113207547	0	53	48	0.9	0.62851	0.988146441	0.371804481	Clonal	.
GCT16T2	ATP6AP1	p.W393*	23	153663826	G	A	Nonsense_Mutation	0.210526316	0.020689655	247	145	1	0.96375	1	0.886216884	Clonal	.
GCT1T	KMT2C	p.Q419R	7	151960144	T	C	Missense_Mutation	0.108108108	0	37	10	0.61	0.40094	0.971822682	0.23354779	Subclonal	.
GCT1T	SYT8	p.R75H	11	1856613	G	A	Missense_Mutation	0.042105263	0	95	72	0.24	0.0009	0.581616864	0.092207265	Subclonal	9.44E-06
GCT1T	ANKRD11	p.G1630R	16	89348062	C	T	Missense_Mutation	0.051612903	0	155	168	0.29	0.00006	0.555131923	0.146653034	Subclonal	.
GCT1T	ZNF419	p.T255A	19	58004685	A	G	Missense_Mutation	0.109589041	0	73	62	0.62	0.31685	0.961612915	0.315217357	Subclonal	.
GCT1T	NRAS	p.G12D	1	115258747	C	T	lissense_Mutation_Hotsp	0.273972603	0	73	71	1	0.89436	1	0.738409194	Clonal	1.88E-05
GCT1T	LY9	p.C320*	1	160784439	C	A	Nonsense_Mutation	0.162790698	0	86	100	0.93	0.68915	1	0.526375369	Clonal	.
GCT1T	ATP6AP2	p.Q188*	23	40457960	C	T	Nonsense_Mutation	0.7	0	10	10	1	0.84018	1	0.602413183	Clonal	.
GCT1T	RNF17	p.X1299_splice	13	25436850	G	T	Splice_Site	0.166666667	0	18	19	0.95	0.60833	0.986504915	0.264122652	Clonal	.
GCT20T2	GRINA	p.F109Lfs*18	8	145065711	G	GC	Frame_Shift_Ins	0.12962963	0	54	25	0.87	0.54493	0.987693878	0.390449577	Clonal	.
GCT20T2	LCORL	p.A22del	4	18023309	AGCG	A	In_Frame_Del	0.086956522	0	23	10	0.58	0.36338	0.97384173	0.151980269	Subclonal	.
GCT20T2	KRTAP5-7	p.G27_S33del	11	71238370	TGGCTCCGGC	A	In_Frame_Del	0.117647059	0	17	12	0.79	0.4709	0.982010353	0.182309339	Subclonal	1.51E-04
GCT20T2	AMOT	p.A926del	23	112022604	GGCA	G	In_Frame_Del	0.0625	0	32	13	0.48	0.28479	0.963994089	0.131878701	Subclonal	.
GCT20T2	CASZ1	p.S783L	1	10713766	G	A	Missense_Mutation	0.078431373	0	51	21	0.53	0.23954	0.956490653	0.203784361	Subclonal	.
GCT20T2	CSF3R	p.R583H	1	36933539	C	T	Missense_Mutation	0.063291139	0	79	35	0.42	0.07106	0.878766928	0.182222149	Subclonal	6.12E-04
GCT20T2	CHDH	p.A79T	3	53857801	C	T	Missense_Mutation	0.235294118	0	34	10	1	0.81331	1	0.547846637	Clonal	.
GCT20T2	GIN1	p.R20Q	5	102444353	C	T	Missense_Mutation	0.061538462	0	65	35	0.41	0.09475	0.897703755	0.161952443	Subclonal	.
GCT20T2	RPP40	p.P304L	6	4995493	G	A	Missense_Mutation	0.051948052	0	77	83	0.35	0.03382	0.815807981	0.136419944	Subclonal	9.42E-06
GCT20T2	ADCY8	p.T1167M	8	131792892	G	A	Missense_Mutation	0.149253731	0	134	83	1	0.71112	1	0.609869638	Clonal	.
GCT20T2	TOPORS	p.S1032L	9	32541428	G	A	Missense_Mutation	0.126984127	0	63	39	0.85	0.53815	0.987394004	0.406547113	Clonal	.
GCT20T2	SPIN1	p.R191C	9	91083502	C	T	Missense_Mutation	0.054347826	0	92	55	0.36	0.02298	0.793185714	0.155968269	Subclonal	.
GCT20T2	OR1B1	p.T285I	9	125390961	G	A	Missense_Mutation	0.053763441	0	93	66	0.36	0.02092	0.786342448	0.154235287	Subclonal	.
GCT20T2	CDC42BPG	p.P380L	11	64605621	G	A	Missense_Mutation	0.078947368	0	38	28	0.53	0.28284	0.964063917	0.178128629	Subclonal	1.07E-05
GCT20T2	ZFHX2	p.S2034Y	14	23993050	G	T	Missense_Mutation	0.075268817	0	93	56	0.5	0.1087	0.91251138	0.245053324	Subclonal	.
GCT20T2	HSPA2	p.T207N	14	65008187	C	A	Missense_Mutation	0.072463768	0	138	75	0.49	0.03358	0.842611155	0.264062625	Subclonal	.
GCT20T2	KIAA1199	p.S805N	15	81218090	G	A	Missense_Mutation	0.052083333	0	96	61	0.35	0.01571	0.765936708	0.149438709	Subclonal	.
GCT20T2	USP36	p.K956R	17	76798561	T	C	Missense_Mutation	0.102564103	0	39	21	0.69	0.40696	0.978670387	0.254064775	Subclonal	.
GCT20T2	COL18A1	p.P1155S	21	46925102	C	T	Missense_Mutation	0.09375	0	32	18	0.63	0.37302	0.97536917	0.203813181	Subclonal	9.58E-06
GCT20T2	ZNF175	p.X24_splice	19	52076655	G	A	Splice_Site	0.095238095	0	42	32	0.64	0.36413	0.974740404	0.240185389	Subclonal	.
GCT2T	ATP6AP1	p.L345Qfs*32	23	153663681	CTCGA	C	Frame_Shift_Del	0.380319149	0	376	431	1	0.89152	1	0.954927697	Clonal	.
GCT2T	SLC35F3	p.V189I	1	234367444	G	A	Missense_Mutation	0.295566502	0.015957447	203	188	1	0.91422	1	0.802130499	Clonal	.
GCT2T	KCNJ3	p.M458L	2	155711691	A	T	Missense_Mutation	0.301886792	0	53	32	1	0.83483	1	0.622763217	Clonal	.
GCT2T	HNRNPDL	p.S17A	4	83350795	A	C	Missense_Mutation	0.42	0	50	43	1	0.88353	1	0.759858122	Clonal	.
GCT2T	SIL1	p.N122S	5	138378397	T	C	Missense_Mutation	0.273584906	0	106	94	0.99	0.83653	1	0.676914192	Clonal	.
GCT2T	PCDHA6	p.P17Q	5	140207726	C	A	Missense_Mutation	0.352941176	0	85	67	1	0.89335	1	0.775079435	Clonal	.
GCT2T	TRIM7	p.L361F	5	180622621	G	A	Missense_Mutation	0.324840764	0	157	270	1	0.92	1	0.81972092	Clonal	.
GCT2T	WRNIP1	p.R211P	6	2766488	G	C	Missense_Mutation	0.160493827	0.020408163	81	49	0.58	0.12278	0.90085206	0.345584804	Subclonal	.
GCT2T	BNC2	p.R536Q	9	16436585	C	T	Missense_Mutation	0.301724138	0	232	172	1	0.9267	1	0.827023621	Clonal	8.47E-05
GCT2T	MARK2	p.D322N	11	63668327	G	A	Missense_Mutation	0.28	0	225	211	1	0.8941	1	0.781811183	Clonal	.
GCT2T	PHGR1	p.H37P	15	40648365	A	C	Missense_Mutation	0.181818182	0	33	54	0.66	0.46733	0.972424101	0.302315783	Subclonal	.
GCT2T	RFFL	p.E313D	17	33339140	C	G	Missense_Mutation	0.339869281	0.01	306	100	1	0.97603	1	0.942582685	Clonal	.
GCT2T	CACNG1	p.L197F	17	65052307	C	T	Missense_Mutation	0.285714286	0	266	422	1	0.91337	1	0.810925477	Clonal	.
GCT2T	PITPNC1	p.E201K	17	65665762	G	A	Missense_Mutation	0.175925926	0	216	129	0.64	0.01229	0.838925128	0.471226987	Subclonal	.
GCT2T	CLASRP	p.R431C	19	45567770	C	T	Missense_Mutation	0.294117647	0	68	89	1	0.84369	1	0.651325578	Clonal	.
GCT2T	C20orf78	p.L73Q	20	18790658	A	T	Missense_Mutation	0.298342541	0	181	120	1	0.91155	1	0.794332956	Clonal	.
GCT2T	RBMXL3	p.C662R	23	114425988	T	C	Missense_Mutation	0.08086785	0.009153318	507	437	0.37	0	0.495339609	0.273856788	Subclonal	8.77E-05
GCT3T	PTCHD2	p.T448I	1	11574473	C	T	Missense_Mutation	0.140495868	0	121	100	1	0.85038	1	0.635207154	Clonal	.
GCT3T	DNAH5	p.V3173M	5	13770946	C	T	Missense_Mutation	0.178571429	0	112	93	1	0.90479	1	0.722943223	Clonal	3.77E-05
GCT3T	RDH10	p.A337T	8	74235254	G	A	Missense_Mutation	0.15625	0	64	64	1	0.84332	1	0.558203987	Clonal	.
GCT3T	EGFL7	p.R112Q	9	139564387	G	A	Missense_Mutation	0.1	0	50	28	0.84	0.65217	0.985976026	0.325710312	Clonal	0.003549
GCT3T	NAT10	p.R966L	11	34165003	G	T	Missense_Mutation	0.159090909	0	44	30	1	0.82698	1	0.48468537	Clonal	.
GCT3T	TENM4	p.H1464Y	11	78413268	G	A	Missense_Mutation	0.126582278	0	158	123	1	0.83561	1	0.630112243	Clonal	.
GCT3T	RBBP8NL	p.A437V	20	60989097	G	A	Missense_Mutation	0.112359551	0	89	59	0.95	0.73806	1	0.472680671	Clonal	.
GCT3T	TTC3	p.W848C	21	38525381	G	T	Missense_Mutation	0.191919192	0	99	82	1	0.92698	1	0.727240501	Clonal	.
GCT3T	KIAA1210	p.R523Q	23	118223625	C	T	Missense_Mutation	0.272222222	0	180	141	1	0.81064	1	0.90115028	Clonal	2.83E-05
GCT3T	ANKRD16	p.Q154*	10	5929885	G	A	Nonsense_Mutation	0.132743363	0	113	68	1	0.82792	1	0.595785965	Clonal	9.42E-06
GCT3T	ATP6AP1	p.Q293*	23	153662746	C	T	Nonsense_Mutation	0.289156627	0	83	69	1	0.82316	1	0.822613583	Clonal	.
GCT4T	ATP6AP2	p.W312fs	23	40464890	GATAAT	G	Frame_Shift_Del	1	0	4	7	1	0.32256	1	0.475491064	Not assessable	.
GCT4T	TPRXL	p.S150T	3	14106124	T	A	Missense_Mutation	0.166666667	0.052631579	42	19	0.56	0.18555	0.934722991	0.274147818	Subclonal	.
GCT4T	A4GNT	p.Y271C	3	137843317	T	C	Missense_Mutation	0.260638298	0.01183432	188	169	0.87	0.62077	0.988705923	0.669920303	Clonal	.
GCT4T	KIAA0947	p.N1423T	5	5463715	A	C	Missense_Mutation	0.349056604	0.017647059	212	170	1	0.91439	1	0.858534635	Clonal	.
GCT4T	COPS6	p.H44R	7	99686967	A	G	Missense_Mutation	0.251396648	0.010526316	179	95	1	0.87088	1	0.770479385	Clonal	.
GCT4T	CEP78	p.T645I	9	80881443	C	T	Missense_Mutation	0.260504202	0	119	104	0.87	0.64073	0.989547848	0.621525856	Clonal	.
GCT4T	UVRAG	p.D658G	11	75852330	A	G	Missense_Mutation	0.365079365	0	126	130	1	0.89188	1	0.821413784	Clonal	.
GCT4T	KBTBD6	p.T403K	13	41705440	G	T	Missense_Mutation	0.052980132	0	151	129	0.18	0	0.33351704	0.086525613	Subclonal	3.77E-05
GCT4T	PYGL	p.E731K	14	51375660	C	T	Missense_Mutation	0.25	0.030769231	36	65	0.84	0.58683	0.986990595	0.432761309	Clonal	9.42E-06
GCT4T	FANCA	p.S1088F	16	89815152	G	A	Missense_Mutation	0.096774194	0	62	51	0.32	0.00143	0.650356072	0.148463503	Subclonal	0.047
GCT4T	RTTN	p.L1708I	18	67721430	G	T	Missense_Mutation	0.253012048	0.014705882	83	68	0.85	0.6017	0.987769662	0.559345436	Clonal	.
GCT5T	PARP12	p.G19Afs*16	7	139762591	GC	G	Frame_Shift_Del	0.095238095	0	21	10	1	0.71557	0.986101942	0.208726388	Clonal	.
GCT5T	SHANK1	p.R493Gfs*70	19	51206832	CG	C	Frame_Shift_Del	0.090909091	0	22	11	1	0.70603	0.985684147	0.205047303	Clonal	.
GCT5T	ATP6AP1	p.T388Sfs*7	23	153663802	TGGCC	T	Frame_Shift_Del	0.148148148	0	270	107	1	0.96652	1	0.860686508	Clonal	.

GCT5T	KMT2A	p.A67del	11	118307413	CGCG	C	In_Frame_Del	0.115384615	0	26	11	1	0.77864	0.989627012	0.298014986	Clonal	.
GCT5T	MPZ	p.M222I	1	161275747	C	G	Missense_Mutation	0.122866894	0	293	106	1	0.87581	1	0.799809723	Clonal	.
GCT5T	NBEAL1	p.E1687A	2	204009621	A	C	Missense_Mutation	0.108333333	0	120	68	1	0.85383	1	0.595703036	Clonal	.
GCT5T	KBTBD8	p.E449K	3	67058348	G	A	Missense_Mutation	0.160714286	0.029411765	56	34	1	0.91894	1	0.589767104	Clonal	2.84E-05
GCT5T	ZNF367	p.R168H	9	99160514	C	T	Missense_Mutation	0.073170732	0	164	90	0.85	0.69769	0.987929767	0.463411038	Clonal	.
GCT5T	ALAD	p.L123Q	9	116153107	A	T	Missense_Mutation	0.108910891	0	101	52	1	0.84605	1	0.563621172	Clonal	.
GCT5T	OR5AK2	p.S291R	11	56757259	A	C	Missense_Mutation	0.392857143	0	28	21	1	0.15723	1	0.708570574	Subclonal	.
GCT5T	MMP7	p.C87R	11	102398564	A	G	Missense_Mutation	0.082524272	0	206	100	0.96	0.78736	1	0.560453134	Clonal	.
GCT5T	C12orf60	p.F21L	12	14975932	T	G	Missense_Mutation	0.24	0	25	19	1	0.86093	1	0.532014402	Clonal	.
GCT5T	MORC4	p.E806K	23	106185412	C	T	Missense_Mutation	0.189873418	0	79	16	1	0.8958	1	0.733819385	Clonal	.
GCT6T	NFKB2	p.G634Dfs*7	10	104160508	AG	A	Frame_Shift_Del	0.211111111	0	90	71	0.91	0.74577	1	0.571812859	Clonal	.
GCT6T	FGR	p.K287Q	1	27942104	T	G	Missense_Mutation	0.212962963	0	216	201	0.92	0.7825	1	0.687155896	Clonal	.
GCT6T	FMN2	p.G941A	1	240370934	G	C	Missense_Mutation	0.43220339	0.016666667	118	60	1	0.96805	1	0.895296215	Clonal	.
GCT6T	SNX17	p.R317C	2	27598547	C	T	Missense_Mutation	0.510373444	0	241	146	1	0.95158	1	0.959214636	Clonal	9.42E-06
GCT6T	IL1B	p.Q257K	2	113587979	G	T	Missense_Mutation	0.179245283	0	106	56	0.77	0.53575	0.979160036	0.49772999	Clonal	.
GCT6T	PLCXD3	p.A113V	5	41382402	G	A	Missense_Mutation	0.209677419	0	62	40	0.9	0.72335	1	0.511598152	Clonal	.
GCT6T	FAM53C	p.R266C	5	137681173	C	T	Missense_Mutation	0.2109375	0.004807692	256	208	0.91	0.76986	1	0.698116212	Clonal	2.83E-05
GCT6T	DIAPH1	p.A325V	5	140958152	G	A	Missense_Mutation	0.304347826	0	46	27	1	0.8574	1	0.643268886	Clonal	1.89E-05
GCT6T	UTRN	p.I2908V	6	145103147	A	G	Missense_Mutation	0.23880597	0	67	49	1	0.80759	1	0.593888127	Clonal	.
GCT6T	ZNF804B	p.I1160N	7	88965775	T	A	Missense_Mutation	0.194285714	0	175	138	1	0.85812	1	0.70415361	Clonal	.
GCT6T	LMOD2	p.D154N	7	123302100	G	A	Missense_Mutation	0.072727273	0	55	27	0.39	0.09781	0.86358698	0.151697087	Subclonal	.
GCT6T	TLE1	p.D750Y	9	84199178	C	A	Missense_Mutation	0.258064516	0	93	85	1	0.86466	1	0.688728976	Clonal	.
GCT6T	DNAJC25	p.I286V	9	114412099	A	G	Missense_Mutation	0.263157895	0	19	20	1	0.8087	1	0.425201438	Clonal	.
GCT6T	PLCE1	p.Q16L	10	95790850	A	T	Missense_Mutation	0.473684211	0	57	43	1	0.9638	1	0.832957184	Clonal	.
GCT6T	PLCE1	p.K18E	10	95790855	A	G	Missense_Mutation	0.491525424	0	59	45	1	0.96158	1	0.845964266	Clonal	.
GCT6T	CNTROB	p.E792V	17	7851640	A	T	Missense_Mutation	0.34562212	0	217	100	1	0.96541	1	0.921100092	Clonal	.
GCT6T	USP32	p.I253T	17	58346864	A	G	Missense_Mutation	0.298245614	0	57	39	1	0.91284	1	0.714136783	Clonal	.
GCT6T	ZNF556	p.T336M	19	2877963	C	T	Missense_Mutation	0.182857143	0	175	82	0.97	0.81961	1	0.668750547	Clonal	1.88E-05
GCT6T	ILVBL	p.P215L	19	15233576	G	A	Missense_Mutation	0.054545455	0	165	77	0.29	0.00001	0.528027506	0.150367832	Subclonal	1.88E-05
GCT6T	ZXDB	p.G206R	23	57619097	G	A	Missense_Mutation	0.052631579	0	76	35	0.38	0.10523	0.869602788	0.150914045	Subclonal	0.001766
GCT6T	PCDH19	p.D412N	23	99662362	C	T	Missense_Mutation	0.140151515	0	264	124	1	0.85488	1	0.70655667	Clonal	.
GCT6T	F8	p.R1671H	23	154157053	C	T	Missense_Mutation	0.255555556	0	90	47	1	0.95309	1	0.784395685	Clonal	.
GCT6T	ATP6AP1	p.W138*	23	153660662	G	A	Nonsense_Mutation	0.563380282	0	213	100	1	0.47717	1	0.964524537	Clonal	.
GCT6T	PPP4R1L		20	56814715	C	T	Splice_Site	0.380434783	0	92	67	1	0.96568	1	0.858922408	Clonal	.
GCT8T	FA2H	p.F254Lfs*27	16	74752909	CG	C	Frame_Shift_Del	0.22	0	100	69	1	0.86119	1	0.735953947	Clonal	.
GCT8T	GPR124	p.G893E	8	37698298	G	A	Missense_Mutation	0.095238095	0	63	31	0.63	0.35935	0.968815685	0.283258531	Subclonal	.
GCT8T	MED13L	p.S635G	12	116446315	T	C	Missense_Mutation	0.048192771	0	166	112	0.32	0.0003	0.60047883	0.158734295	Subclonal	.
GCT8T	ATP6V0C	p.E139K	16	2569693	G	A	Missense_Mutation	0.18490566	0	265	165	1	0.88654	1	0.808224612	Clonal	.
GCT8T	CYLC1	p.K201T	23	83128318	A	C	Missense_Mutation	0.230769231	0	39	23	1	0.88247	1	0.588119027	Clonal	.
GCT92T	SLC7A4	p.G236Afs*55	22	21385396	CG	C	Frame_Shift_Del	0.204819277	0	83	98	1	0.93854	1	0.744437573	Clonal	.
GCT92T	CLASP2	p.M965Wfs*33	3	33602360	AT	A	Frame_Shift_Del	0.057692308	0	52	74	0.62	0.49097	0.974709329	0.199193299	Subclonal	1.89E-05
GCT92T	SPTA1	p.L1370Afs*10	1	158615065	C	CT	Frame_Shift_Ins	0.085714286	0	35	53	0.91	0.65276	0.985775524	0.254452732	Clonal	.
GCT92T	OR7C1	p.T105Hfs*17	19	14910637	G	GA	Frame_Shift_Ins	0.114285714	0.022727273	70	88	1	0.79746	1	0.491034335	Clonal	.
GCT92T	OSGEPL1	p.K13*	2	190626330	T	TA	Frame_Shift_Ins	0.081081081	0	37	47	0.86	0.63444	0.984863471	0.247166534	Clonal	.
GCT92T	TYSND1	p.L138del	10	71905928	TCAG	T	In_Frame_Del	0.053333333	0	75	74	0.57	0.41422	0.966707264	0.216951104	Subclonal	6.22E-04
GCT92T	PPP1R9B	p.P257del	17	48227102	GGGC	G	In_Frame_Del	0.050847458	0	59	59	0.54	0.41929	0.967015618	0.180732323	Subclonal	1.76E-04
GCT92T	NCL	p.G698del	2	232319941	ACCT	A	In_Frame_Del	0.050847458	0	59	72	0.54	0.41929	0.967015618	0.180732323	Subclonal	1.88E-05
GCT92T	HNF4A	p.L341del	20	43052772	AGCT	A	In_Frame_Del	0.050632911	0	79	106	0.54	0.375	0.961355146	0.207527791	Subclonal	0.004049
GCT92T	DBNDD2	p.S223del	20	44038637	ACCT	A	In_Frame_Del	0.093023256	0	43	49	0.99	0.69067	0.988150744	0.315248405	Clonal	0.001643
GCT92T	MYH9	p.E1350del	22	36689418	GCCT	G	In_Frame_Del	0.052631579	0	76	91	0.56	0.40443	0.965461092	0.214514477	Subclonal	1.32E-04
GCT92T	CARD10	p.K272_E273del	22	37906308	GCTCCTT	G	In_Frame_Del	0.075	0	40	49	0.8	0.60675	0.983336304	0.236595533	Clonal	.
GCT92T	FGF10	p.C23del	5	44388714	AAGC	A	In_Frame_Del	0.052631579	0	76	104	0.56	0.40443	0.965461092	0.214514477	Subclonal	4.71E-04
GCT92T	C6orf132	p.L176_P184del	6	42075104	GTTCCAGCAGC	G	In_Frame_Del	0.25	0	8	7	1	0.76869	1	0.258968278	Clonal	0.018
GCT92T	ARID1B	p.Q131del	6	157099426	ACAG	A	In_Frame_Del	0.068627451	0.011235955	102	89	0.73	0.55733	0.980835923	0.34086242	Clonal	.
GCT92T	MLLT3	p.S190del	9	20414343	ACTG	A	In_Frame_Del	0.125	0	16	13	1	0.72074	0.987498656	0.222715531	Clonal	.
GCT92T	RBM10	p.R85del	23	47030466	AGGC	A	In_Frame_Del	0.055555556	0.007194245	90	139	0.59	0.41535	0.967062763	0.248632975	Subclonal	5.10E-04
GCT92T	CYP4Z1	p.R374C	1	47571852	C	T	Missense_Mutation	0.111111111	0	27	37	1	0.72568	0.988743094	0.28572146	Clonal	7.53E-05
GCT92T	TET1	p.S879L	10	70405122	C	T	Missense_Mutation	0.090909091	0	44	72	0.97	0.68303	0.987821916	0.311617297	Clonal	9.42E-06
GCT92T	CCDC92	p.E306K	12	124421685	C	T	Missense_Mutation	0.121212121	0	33	44	1	0.76715	1	0.353850049	Clonal	3.77E-05
GCT92T	THSD4	p.L733H	15	72039338	T	A	Missense_Mutation	0.255319149	0	47	52	1	0.85954	1	0.69661376	Clonal	.
GCT92T	HS3ST2	p.T216M	16	22926426	C	T	Missense_Mutation	0.119496855	0.005181347	159	193	1	0.86219	1	0.671710308	Clonal	1.88E-05
GCT92T	IGF2BP1	p.N353I	17	47119720	A	T	Missense_Mutation	0.212765957	0	47	79	1	0.91503	1	0.639108808	Clonal	.
GCT92T	CEP44	p.R11Q	4	175220304	G	A	Missense_Mutation	0.0625	0	64	82	0.67	0.5185	0.977455992	0.246084011	Clonal	4.14E-04
GCT92T	P4HA2	p.E438K	5	131533958	C	T	Missense_Mutation	0.102564103	0	39	67	1	0.72119	0.98934767	0.330402832	Clonal	.
GCT92T	ALKBH4	p.S205L	7	102098136	G	A	Missense_Mutation	0.078431373	0	51	69	0.84	0.62871	0.985117548	0.286856799	Clonal	.
GCT92T	MSR1	p.V387I	8	15977990	C	T	Missense_Mutation	0.25	0	80	109	1	0.85505	1	0.791132929	Clonal	9.42E-06
GCT92T	DIAPH2	p.P558Q	23	96212885	C	A	Missense_Mutation	0.204081633	0	49	49	1	0.91941	1	0.634403319	Clonal	.
GCT92T	ATP6AP1	p.W302*	23	153662775	G	A	Nonsense_Mutation	0.25	0.02173913	48	46	1	0.87116	1	0.694800345	Clonal	.
GCT9T	ATP6AP2	p.P90Qfs*48	23	40450581	AC	A	Frame_Shift_Del	0.382352941	0	68	56	1	0.95367	1	0.819038354	Clonal	.
GCT9T	RNF123	p.L608Pfs*12	3	49740923	T	TG	Frame_Shift_Ins	0.160493827	0	162	182	0.67	0.10316	0.920749165	0.464674723	Subclonal	9.42E-06
GCT9T	INSRR	p.A1011D	1	156812890	G	T	Missense_Mutation	0.27027027	0	111	120	1	0.83874	1	0.730685855	Clonal	.
GCT9T	GRHL1	p.T272M	2	10104083	C	T	Missense_Mutation	0.145454545	0	55	47	0.61	0.24783	0.955280778	0.311472006	Subclonal	.
GCT9T	ALS2CL	p.Q206K	3	46727848	G	T	Missense_Mutation	0.169230769	0	65	64	0.71	0.37727	0.973922216	0.39811213	Subclonal	.
GCT9T	GPER1	p.T66I	7	1131561	C	T	Missense_Mutation	0.198412698	0	252	360	0.83	0.45979	0.981590639	0.637969086	Subclonal	.
GCT9T	OR5B2	p.S62R	11	58190549	A	T	Missense_Mutation	0.178082192	0	146	103	0.75	0.30503	0.967023891	0.515823525	Subclonal	.
GCT9T	PLBD1	p.L202V	12	14689599	G	C	Missense_Mutation	0.134328358	0	67	46	0.56	0.13906	0.924498063	0.299468357	Subclonal	.
GCT9T	DGKA	p.E278K	12	56334131	G	A	Missense_Mutation	0.232067511	0.003333333	237	300	0.97	0.79526	1	0.740210112	Clonal	.
GCT9T	LTA4H	p.A173T	12	96415993	C	T	Missense_Mutation	0.266666667	0	60	43	1	0.77418	1	0.623669874	Clonal	.
GCT9T	AMER2	p.I362F	13	25744674	T	A	Missense_Mutation	0.25443787	0	169	221	1	0.84564	1	0.75577534	Clonal	.

Supplementary Table 5: Histologic features of granular cell tumors according to *ATP6AP1* and *ATP6AP2* mutational status.

Fanburg-Smith histologic criteria		*ATP6AP1*^{WT}/*ATP6AP2*^{WT} (n=23)	*ATP6AP1*^{MUT} (n=50)	*ATP6AP2*^{MUT} (n=9)	p value[a]
Necrosis	No	23 (100%)	48 (96%)	9 (100%)	0.999
	Yes	0 (0%)	2 (4%)	0 (0%)	
Spindling	No	16 (69.6%)	34 (68%)	7 (77.8%)	0.939
	Yes	7 (30.4%)	16 (32%)	2 (22.2%)	
Vesicular nuclei and prominent nucleoli	No	20 (87%)	36 (72%)	8 (88.9%)	0.344
	Yes	3 (13%)	14 (28%)	1 (11.1%)	
Mitoses/10 HPF	0-2	21 (91.3%)	47 (94%)	9 (100%)	0.806
	>2	2 (8.7%)	3 (6%)	0 (0%)	
Increased nuclear/ cytoplasmic ratio	No	21 (91.3%)	46 (92%)	8 (88.9%)	0.859
	Yes	2 (8.7%)	4 (8%)	1 (11.1%)	
Nuclear pleomorphism	No	21 (91.3%)	39 (78%)	9 (100%)	0.181
	Yes	2 (8.7%)	11 (22%)	0 (0%)	
Fanburg-Smith histologic category	Benign	14 (60.9%)	24 (48%)	6 (66.7%)	0.787
	Atypical	8 (34.8%)	21 (42%)	3 (33.3%)	
	Malignant	1 (4.3%)	5 (10%)	0 (0%)	

[a]Fisher's exact test. HPF, high power fields; MUT, mutant; WT, wild-type

SUPPLEMENTARY REFERENCES

1. Cancer Genome Atlas Research Network*, et al.* Genomic and epigenomic landscapes of adult de novo acute myeloid leukemia. *N Engl J Med* **368**, 2059-2074 (2013).
2. Cancer Genome Atlas Research Network. Comprehensive molecular characterization of urothelial bladder carcinoma. *Nature* **507**, 315-322 (2014).
3. Ciriello G*, et al.* Comprehensive Molecular Portraits of Invasive Lobular Breast Cancer. *Cell* **163**, 506-519 (2015).
4. Cancer Genome Atlas Network. Comprehensive molecular characterization of human colon and rectal cancer. *Nature* **487**, 330-337 (2012).
5. Cancer Genome Atlas Network. Comprehensive genomic characterization of head and neck squamous cell carcinomas. *Nature* **517**, 576-582 (2015).
6. Cancer Genome Atlas Research Network. Comprehensive molecular characterization of clear cell renal cell carcinoma. *Nature* **499**, 43-49 (2013).
7. Davis CF*, et al.* The somatic genomic landscape of chromophobe renal cell carcinoma. *Cancer Cell* **26**, 319-330 (2014).
8. Ceccarelli M*, et al.* Molecular Profiling Reveals Biologically Discrete Subsets and Pathways of Progression in Diffuse Glioma. *Cell* **164**, 550-563 (2016).
9. Campbell JD*, et al.* Distinct patterns of somatic genome alterations in lung adenocarcinomas and squamous cell carcinomas. *Nat Genet* **48**, 607-616 (2016).
10. Cancer Genome Atlas Research Network. Integrated genomic analyses of ovarian carcinoma. *Nature* **474**, 609-615 (2011).
11. Cancer Genome Atlas Research Network. The Molecular Taxonomy of Primary Prostate Cancer. *Cell* **163**, 1011-1025 (2015).
12. Cancer Genome Atlas Research Network. Comprehensive molecular characterization of gastric adenocarcinoma. *Nature* **513**, 202-209 (2014).

13. Cancer Genome Atlas Research Network*, et al.* Integrated genomic characterization of endometrial carcinoma. *Nature* **497**, 67-73 (2013).

14. Cancer Genome Atlas Research Network. Integrated genomic characterization of papillary thyroid carcinoma. *Cell* **159**, 676-690 (2014).
